# Beta burst dynamics in Parkinson’s disease OFF and ON dopaminergic medication

**DOI:** 10.1093/brain/awx252

**Published:** 2017-11-01

**Authors:** Gerd Tinkhauser, Alek Pogosyan, Huiling Tan, Damian M. Herz, Andrea A. Kühn, Peter Brown

**Affiliations:** 1 Medical Research Council Brain Network Dynamics Unit at the University of Oxford, Oxford, UK; 2 Nuffield Department of Clinical Neurosciences, John Radcliffe Hospital, University of Oxford, Oxford, UK; 3 Department of Neurology, Bern University Hospital and University of Bern, Switzerland; 4 Department of Neurology, Charitè, Universitätsmedizin Berlin, Germany

**Keywords:** Parkinson’s disease, beta oscillations, basal ganglia, closed-loop control, levodopa

## Abstract

Exaggerated basal ganglia beta activity (13–35 Hz) is commonly found in patients with Parkinson’s disease and can be suppressed by dopaminergic medication, with the degree of suppression being correlated with the improvement in motor symptoms. Importantly, beta activity is not continuously elevated, but fluctuates to give beta bursts. The percentage number of longer beta bursts in a given interval is positively correlated with clinical impairment in Parkinson’s disease patients. Here we determine whether the characteristics of beta bursts are dependent on dopaminergic state. Local field potentials were recorded from the subthalamic nucleus of eight Parkinson’s disease patients during temporary lead externalization during surgery for deep brain stimulation. The recordings took place with the patient quietly seated following overnight withdrawal of levodopa and after administration of levodopa. Beta bursts were defined by applying a common amplitude threshold and burst characteristics were compared between the two drug conditions. The amplitude of beta bursts, indicative of the degree of local neural synchronization, progressively increased with burst duration. Treatment with levodopa limited this evolution leading to a relative increase of shorter, lower amplitude bursts. Synchronization, however, was not limited to local neural populations during bursts, but also, when such bursts were cotemporaneous across the hemispheres, was evidenced by bilateral phase synchronization. The probability of beta bursts and the proportion of cotemporaneous bursts were reduced by levodopa. The percentage number of longer beta bursts in a given interval was positively related to motor impairment, while the opposite was true for the percentage number of short duration beta bursts. Importantly, the decrease in burst duration was also correlated with the motor improvement. In conclusion, we demonstrate that long duration beta bursts are associated with an increase in local and interhemispheric synchronization. This may compromise information coding capacity and thereby motor processing. Dopaminergic activity limits this uncontrolled beta synchronization by terminating long duration beta bursts, with positive consequences on network state and motor symptoms.

## Introduction

Basal ganglia beta activity (13–35 Hz) is well known to be exaggerated in patients with Parkinson’s disease, and the amplitude of such activity has been linked to motor impairment (Brown, 2003) and dopaminergic tone (Jenkinson and Brown, 2011). In particular, the reduction in beta power in the local field potential (LFP) recorded in the subthalamic nucleus (STN) after administration of levodopa and during continuous high frequency deep brain stimulation (DBS) is positively correlated with improvement of motor impairment (Kühn *et al.*, 2006, 2008, 2009; Chen *et al.*, 2010; López-Azcárate *et al.*, 2010; Eusebio *et al.*, 2011; Özkurt *et al.*, 2011; Oswal *et al.*, 2016; Trager *et al.*, 2016). As such, beta activity in the STN has been used as a feedback signal in amplitude-responsive closed-loop DBS, where stimulation is delivered when beta amplitude rises above a certain threshold (Little *et al.*, 2013*a*) or in proportion to beta amplitude (Rosa *et al.*, 2015, 2017). Initial, albeit acute, studies have suggested that this adaptive approach can be at least as effective as conventional, continuous DBS, while using less battery power and incurring fewer stimulation-induced side effects, such as speech impairment and dyskinesias (Little *et al.*, 2013*a*, 2016*a*, *b*; Rosa *et al.*, 2015, 2017; Pina-Fuentes *et al.*, 2017).

However, one unresolved aspect of the pathological exaggeration of beta activity in Parkinson’s disease that impacts on the delivery of adaptive DBS is whether beta activity is tonically or phasically elevated. Evidence is beginning to accrue that physiological beta activity consists of short-lived phasic bursts in basal ganglia-cortical motor circuits (Murthy and Fetz, 1992, 1996; Feingold *et al.*, 2015) and studies in Parkinson’s disease patients undergoing DBS suggest that pathological beta activity may tend to consist of longer duration, phasic bursts (Tinkhauser *et al.*, 2017). Adaptive DBS has therefore been suggested to selectively trim longer beta bursts leading to a redistribution of beta bursts towards shorter, more physiological, durations (Tinkhauser *et al.*, 2017). Here we test the hypotheses that pathological beta activity consists of prolonged bursts in Parkinson’s disease, that these bursts are associated with excessive synchronization within and between basal ganglia circuits and that such bursts are abbreviated and made less frequent by treatment with the dopaminergic prodrug, levodopa, thereby contributing to improved motor function.

## Materials and methods

### Subjects and surgery

We investigated beta bursts before and after administration of levodopa in eight patients (16 hemispheres) with advanced Parkinson’s disease undergoing DBS surgery targeting the STN (Table 1). All subjects have been previously reported (Kühn *et al.*, 2006). They gave their written informed consent and the local ethics committee approved the study. Inclusion criteria for each hemisphere were the presence of a beta peak in the OFF levodopa condition and a minimum recording duration of 2 min of artefact-free signal. Consequently, one subject from the original study was not included.

### Experiments and recordings

DBS electrodes were temporarily externalized prior to connection to the implantable pulse generator. LFP recordings were performed with the patient quietly seated following overnight withdrawal of antiparkinsonian medication before and after administration of levodopa (test challenge dose in Table 1) 3–6 days after lead implantation. In four subjects (Subjects 1, 2, 6 and 8) LFPs were also recorded during self-paced, discrete, front-and-back joystick movements; LFPs were recorded from adjacent bipolar contact pairs (01, 12, 23) and the contact pair with the highest beta power in the OFF condition was selected for further analyses. Signals were amplified and filtered at 1–250 Hz using a custom-made, high-impedance amplifier (which had at its front end input stage the INA 128 instrumentation amplifier, Texas Instruments) and recorded through a 1401 analogue/digital converter (Cambridge Electronic Design) onto a computer using Spike2 software (Cambridge Electronic Design). Signals were sampled at either 625 Hz or 1 kHz. Before and after administration of levodopa, motor symptoms were assessed using the Unified Parkinson’s Disease Rating Scale (UPDRS). Half points were used to increase the sensitivity of the test.

### Signal processing and determination of bursts

**Figure 1:**
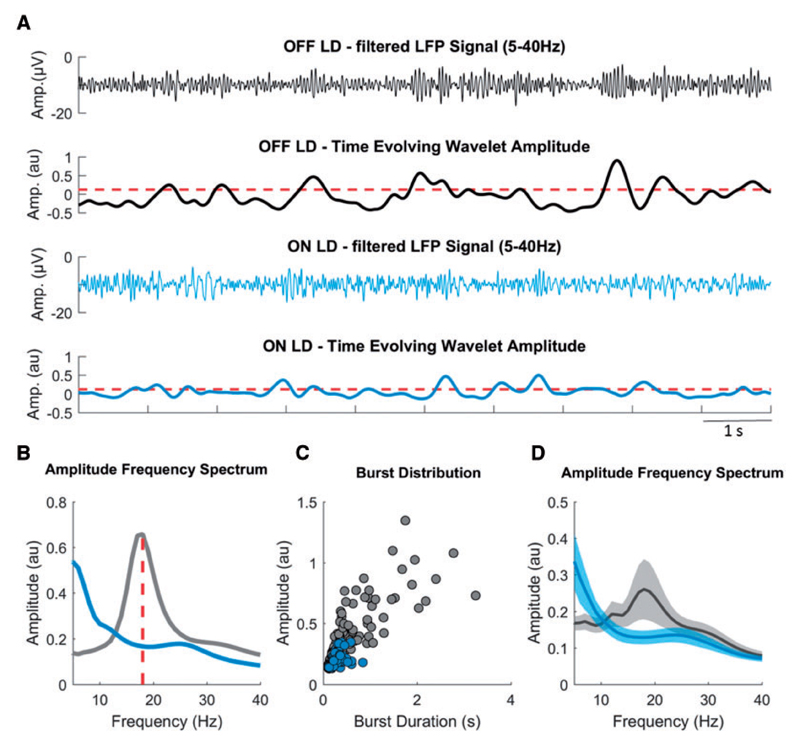
Burst determination. (**A**) A segment of the filtered LFP signal (5–40 Hz) and the time evolving wavelet amplitude (from the same segment) of the beta peak frequency (18 Hz) derived from the wavelet transformed signal, both for OFF (grey) and ON (blue) levodopa. The red dashed horizontal line illustrates the common amplitude threshold, which corresponds to the mean of the 75th percentile amplitudes of OFF and ON levodopa. Periods of the time evolving wavelet amplitude that cross this threshold for longer than 0.1 s were defined as beta bursts. (**B**) The LFP amplitude spectra for OFF and ON levodopa, with a beta peak at 18 Hz in the OFF levodopa condition and reduction of beta amplitude in the ON levodopa condition. (**C**) Amplitude and duration of all detected beta bursts for both the conditions taken from recordings of 258 s and 318 s duration. Example Subject 3, right side. (**D**) The average LFP amplitude spectra across all hemispheres for OFF and ON levodopa, with the reduction of beta amplitude during the ON levodopa condition. Values are represented as mean + SEM. LD = levodopa.

Figure 1 and Supplementary Fig. 1 illustrate the processing steps involved in the discrimination of bursts of beta activity. After visual signal inspection and artefact removal using Spike2 Software, the data were imported into Matlab (version R 2015b; MathWorks, Natick, MA), where all further signal processing steps took place. Signal duration ranged from 136 s to 365 s with a mean signal duration of 232.0 ± 14.2 s for the OFF condition and 231.9 ± 15.5 s for the ON condition without significant difference [*t*(15) = 0.006, *P* = 0.995]. Signal durations between left and right STNs were matched in both OFF and ON conditions.

The signal was resampled at 300 Hz, highpass filtered at 1 Hz and decomposed using Wavelet transformation (ft_specest_wavelet script in Fieldtrip - Morlet Wavelet, width = 10, gwidth = 5; Donders Institute for Brain, Cognition and Behaviour, 2010) into frequency components between 1 and 40 Hz with the frequency resolution of 1 Hz. The beta peak frequency (single frequency bin of 1 Hz) was selected in the OFF levodopa state and the corresponding time evolved wavelet amplitude was smoothed (0.2 s) and DC corrected (20 s time constant) to adjust for potential baseline shifts in amplitude, such as those due to variable amplifier noise floor, and to focus on the variance in beta amplitude. The determination of bursts followed the same algorithm used and justified in our previous study (Tinkhauser *et al.*, 2017) and is summarized below.

The duration of beta bursts was determined by the time points at which the selected time evolved wavelet amplitude exceeded a given amplitude threshold. Thresholds were defined in terms of percentiles of the DC corrected signal amplitude distribution. However, as the precise amplitudes of percentile-defined thresholds could vary between ON and OFF conditions, the applied threshold was set as the average of the amplitudes corresponding to the selected percentile, and the same threshold applied to both the conditions for a given hemisphere. Thus when the text refers to, for example, thresholding according to the 75th percentile, the same threshold equivalent to the mean of the 75th percentiles across conditions, was applied to each condition, unless otherwise stated.

The selection of a given percentile amplitude threshold to determine bursts is somewhat arbitrary, although previous work has shown that relative differences in burst properties during different conditions (no stimulation, adaptive DBS and conventional DBS) were preserved across various amplitude thresholds. To investigate if the same is true for beta bursts OFF and ON levodopa, we additionally defined them using a range of different percentile thresholds (55, 60, 65, 70, 75, 80, 85, 90 percentiles) and include these data in our results.

In general, we did not consider bursts with durations shorter than 100 ms (less that about two cycles in duration) to limit the contribution of spontaneous fluctuations in amplitude due to noise. The distribution of burst durations was considered by categorizing them into nine time windows of 100 ms starting from 100 ms to >900 ms in duration (Fig. 1). However, for illustrative purposes we have also included bursts with very short durations between 0.05 s and 0.1 s as a separate time window in Supplementary Fig. 2. Note that the last time window (>900 ms) includes bursts with a broader range of duration. This was necessary as longer bursts became progressively less frequent and like this we ensured sufficient burst numbers in each window. Since the total signal duration could vary between subjects, instead of the absolute number of bursts per time window, the percentage distribution of bursts was chosen. Burst duration is also illustrated as mean burst duration without prior categorization into time windows.

As noted above, the smoothed and DC-removed time evolved wavelet amplitude of the beta peak frequency served as a basis to determine beta bursts. However, to exclude the possibility that shifts in the beta peak frequency over time or between OFF and ON conditions affected the burst distribution, we repeated key analyses with a more relaxed beta peak definition (beta peak ± 5 Hz). Furthermore, to illustrate an amplitude independent estimate of the burst dynamics we calculated a wavelet-based frequency distribution (0.2 Hz to 2 Hz with frequency resolution of 0.1 Hz) of the time evolved beta amplitude (same as used for burst segmentation) for both the conditions.

For the recordings with self-paced joystick movements, determination of beta bursts was performed as described previously, with a common 75th percentile amplitude threshold. The total events per hemisphere were quantified and burst characteristics, including burst duration and burst probability (bursts/s), where derived from the 3 s before movement onset and 1 s after movement onset (during movement).

### Data analysis and statistics

The burst results were derived and compared at the level of each hemisphere. To investigate the distribution of bursts with different durations between the conditions, we performed a two-way repeated measurement analysis of variance (ANOVA) with a 9 × 2 design (nine time windows, two conditions). Additionally, for each hemisphere the average burst duration, the average burst amplitude, burst probability (bursts/s), percentage time spent as burst as well as the burst probability and burst duration before and during movement were calculated and compared.

The relationship between burst duration and burst amplitude was calculated by applying a Spearman bivariate correlation on all detected bursts (rs = Spearman’s rho) for each hemisphere and condition and values were Fisher transformed before averaging. Slopes between the conditions were compared using the non-standardized coefficient of the first order fit (change in amplitude per time unit).

To compare burst dynamics between hemispheres, we first calculated the percentage number of beta bursts in a given interval that were overlapping between the left and right hemisphere for both the conditions at the frequency of the beta peak. Since the beta peak frequency between the left and right hemisphere can vary (Table 1), the percentage overlapping was calculated for both hemispheric beta peak frequencies and then averaged. The same algorithm was then repeated for the 10 neighbouring frequency bins on the left and right of the peak beta frequency. The coupling between bursts was investigated using the phase synchrony index (PSI) and compared between related-overlapping (naturally co-occurring) bursts and unrelated-overlapping, shuffled burst periods. Since the duration of overlapping bursts varied, we only considered the central overlapping 200 ms of every burst. The activities on the two sides during this period of bursting were independently concatenated to give two time series, either in correct order or in a bilaterally independently shuffled order. The PSI was then calculated. Accordingly, overlapping bursts with <200 ms duration overlap did not contribute to PSI estimates. Similar to the calculation of the percentage burst overlapping between STNs, the PSI was calculated for peak beta frequencies in both hemispheres and then averaged, since the beta peak frequency between the left and right hemisphere could vary (Table 1). The PSI was calculated according to following formula, in which *n* is the number of time points, and *φ*_STN-Le_ is the phase angle for the left STN and *φ*_STN-Ri_ the phase angle for the right hemisphere.

(1) $$PSI=n^{-1}\sum_{t=1}^{n}e^{-i(\varphi_{STN-Le}-\varphi_{STN-Ri})}$$

Key results such as burst duration, burst amplitude, and percentage burst overlapping were not only compared between ON and OFF state, but also to results obtained by chance. This was achieved by shuffling of the raw LFP signal (1000 permutations) and thereafter by applying the same burst determination algorithm.

For the clinical correlations, we first used the percentage number of beta bursts in a given interval for each binned burst duration and correlated them with clinical impairment, to determine the overall relationship between burst duration and motor performance.

The motor performance was given by the sum of the hemibody UPDRS Part III items (items 20 to 26), and separately also as the sum of key sub-items (bradykinesia, rigidity and tremor), contralateral to the side of LFP recording and bursts considered as the percentage of short bursts and long bursts relative to all the bursts from 100 ms to >900 ms.

For the relationship between the change of burst duration and clinical improvement we correlated the ratio between burst duration OFF levodopa and ON levodopa for the peak frequency and the neighbouring frequencies (± 10 Hz) with the clinical improvement. The latter was derived using the following formula:

(2) 100×(hemibodyscoreOFF-med−hemibodyscoreON-med)/hemibodyscoreOFF-med

Statistical analyses were performed using IBM SPSS Statistics Version 23. All data are presented as means ± standard error of the mean (SEM), unless otherwise stated. The assumption of a normal distribution was tested by visual inspection of the QQ-plots. Pairwise comparisons of burst parameters were performed with paired *t*-tests. An exception was the burst analysis during movement, where because of the low sample size (eight hemispheres) non-parametric statistics (Wilcoxon signed rank test) were applied and z-scores, pairs of comparisons and positive and negative ranks reported. If for the repeated measures ANOVAs Mauchly’s test indicated that the assumption of sphericity was violated, Greenhouse-Geisser corrections were applied. To evaluate statistical significance between the conditions for burst duration and percentage overlapping across a selection of frequencies (beta peak frequency ± 10 Hz) we used a cluster-based permutation procedure: *P*-values were derived by randomly permuting the assignment of condition labels for all hemispheres/subjects 1000 times. For each frequency point the z-statistic of the actual mean difference was computed based on the distribution of the 1000 differences resulting from permutation. The resulting *P*-values were then corrected for multiple comparisons using a cluster-based permutation approach. Then suprathreshold clusters (pre-cluster threshold: *P* < 0.05) were determined for each permutation, and the sum of the z-statistics within these clusters was stored to form a distribution of the largest suprathreshold-cluster values. Finally, the 95th percentile of this distribution served as statistical threshold for the map of the actual z-statistics (Maris and Oostenveld, 2007). Clinical correlations were performed using Spearman’s correlation. For the comparison between clinical improvement and ratio in burst duration between OFF and ON levodopa, an additional bootstrap method was used to determine the 95th confidence interval of the correlation coefficients for each frequency bin. To control for multiple comparisons we performed the false discovery rate (FDR) correction procedure, which controls the expected proportion of falsely rejected hypotheses (Benajmini and Hochberg, 1995).

## Results

### Relative burst duration distribution differs during OFF and ON levodopa

**Figure 2:**
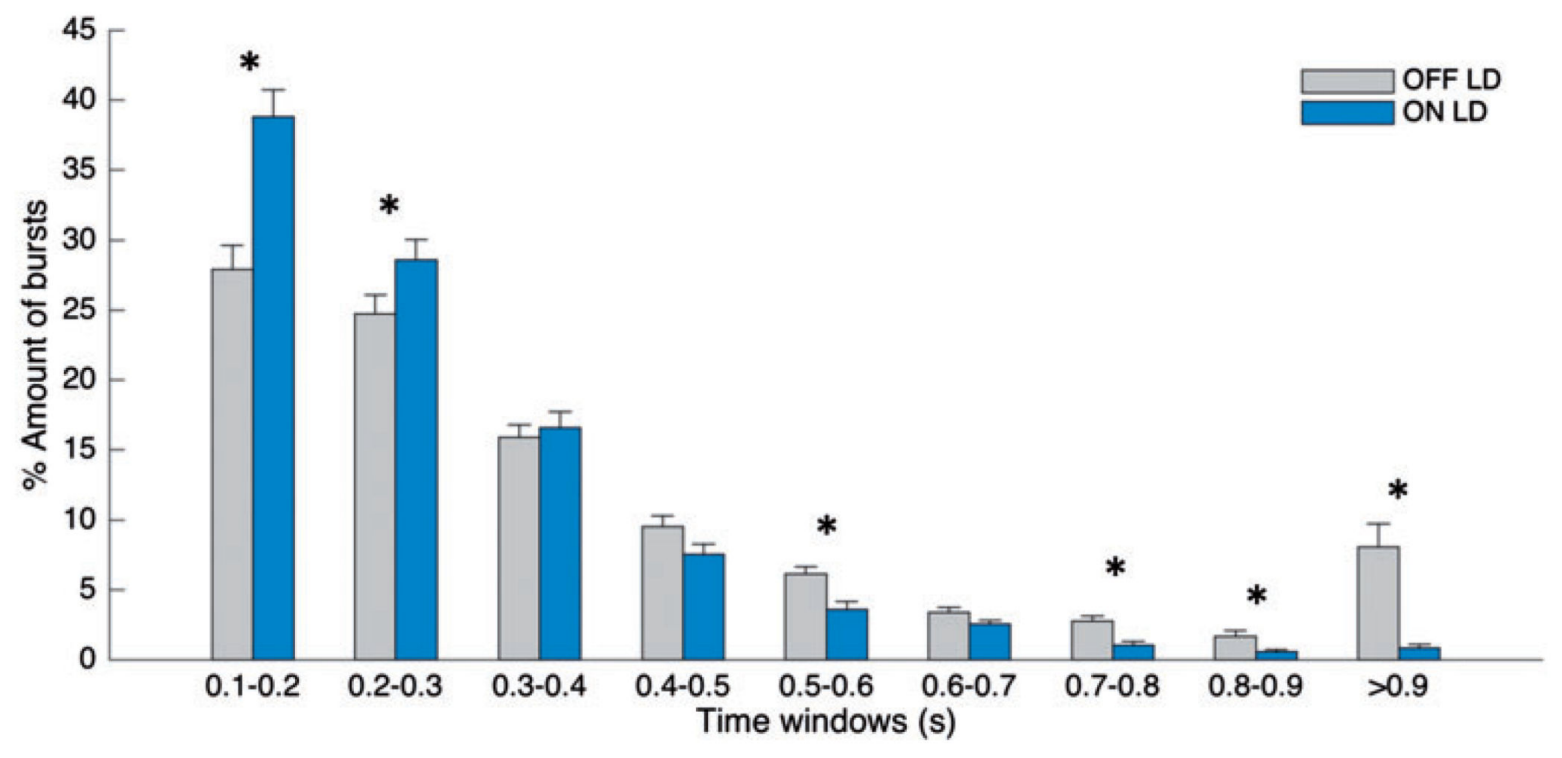
Change in burst duration distribution. Distribution of burst durations averaged across 16 sides as a percentage of total number of bursts on each side, during OFF levodopa and ON levodopa, where bursts are defined as periods of beta activity that exceed the 75th percentile amplitude threshold with a minimum duration of 0.1 s. During ON levodopa the percentage amount of shorter bursts (0.1–0.2 s, 0.2–0.3 s) is higher and the percentage amount of long bursts (0.5–0.6 s, 0.7–0.8 s, 0.8–0.9 s, >0.9 s) is lower in comparison to the OFF levodopa state. Values are represented as mean + SEM; *P_corr_ < 0.05. LD = levodopa.

Figure 2 illustrates the percentage distribution of burst durations across different burst time windows (bins) and conditions for the 75th percentile amplitude threshold. A repeated measures ANOVA showed a significant main effect for the interaction between condition and burst duration [*F*(df 2.333) = 12.932, *P* < 0.001]. The corresponding *post hoc* comparison between OFF levodopa and ON levodopa showed that the percentage number of shorter beta bursts (0.1–0.2 s; 0.2–0.3 s) in a given interval was higher during ON levodopa compared to OFF levodopa [*t*(15) = −4.257 *P* = 0.002, *t*(15) = −2.38 *P* = 0.047]. In contrast, the percentage number of longer bursts (0.5–0.6 s; 0.7–0.8 s; 0.8–0.9 s; >0.9 s) was higher during OFF levodopa compared to ON levodopa [*t*(15) = 14.39, *P* = 0.002; *t*(15) = 4.93, *P* < 0.001; *t*(15) = 2.51, *P* = 0.043; *t*(15) = 3.96, *P* = 0.002]. The lack of significant difference between OFF and ON levodopa for the time windows 0.3–0.5 is because of the averaging across hemispheres. Individual hemispheres show a transition effect (from short bursts being relatively preferred ON drug to long bursts being relatively preferred OFF drug) over 0.3–0.5 s, but as the precise transition point varies a little between hemispheres there is no significant change in these bins. Supplementary Fig. 2 illustrates the relative burst distribution including bursts with duration shorter than 0.1 s (bin range 0.05–0.1 s). These very short bursts show a similar pattern, being significantly more common during ON levodopa compared to OFF levodopa, although the total number of these bursts is smaller compared to that in the neighbouring bin (0.1–0.2 s).

### Burst duration and amplitude is reduced ON levodopa

**Figure 3:**
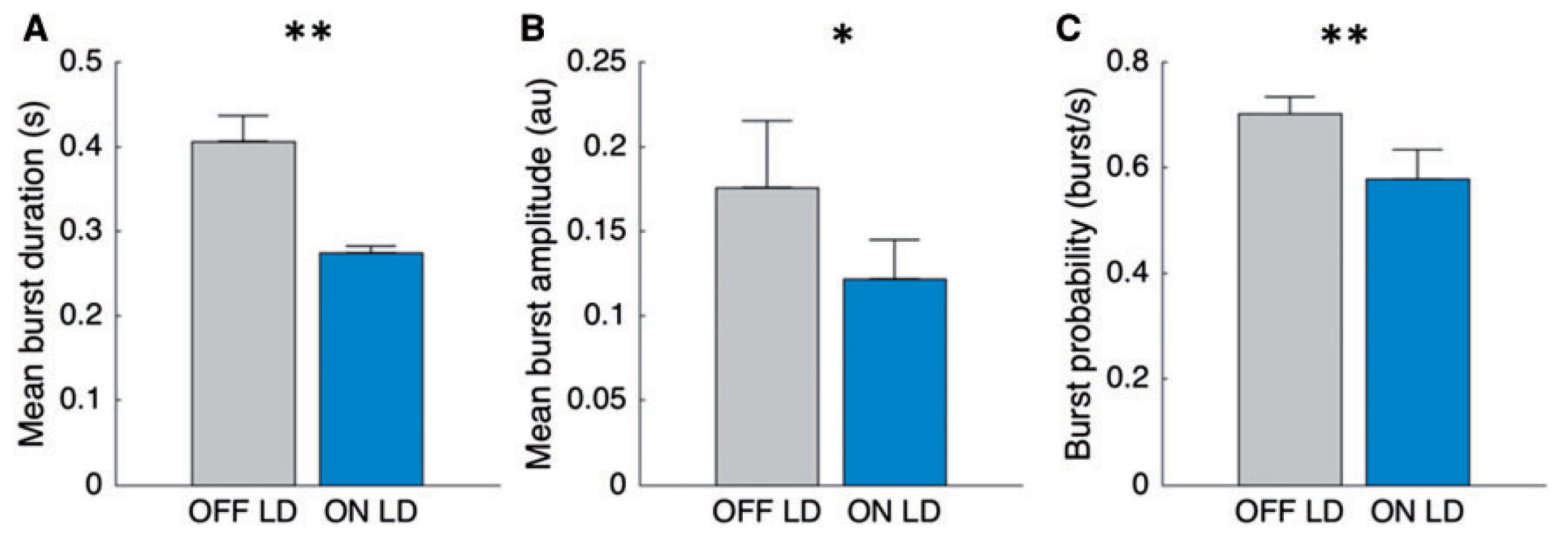
Change in burst duration, burst amplitude and burst probability. (**A**) The mean burst duration, derived from the mean duration in each STN (without prior categorization into burst time windows) during OFF and ON levodopa. During ON levodopa, mean burst duration is significantly reduced in comparison to OFF levodopa. The same significant differences were seen when we compare the average of the individual median burst durations across conditions [0.297 s ± 0.015 versus 0.234 s ± 0.008; *t*(15) = 3.62, *P* = 0.003]. (**B**) The mean burst amplitude during OFF and ON levodopa. There is a significant reduction in burst amplitude after administration of levodopa. (**C**) The probability of bursts to occur (illustrated as burst/s) is reduced after administration of levodopa. Beta bursts were determined using the 75th percentile amplitude threshold. Values are represented as mean + SEM; *P < 0.05, **P < 0.01. LD = levodopa.

How does the duration of bursts change between conditions without prior categorization of burst durations into burst time windows? Figure 3A illustrates the difference in mean burst duration before (0.406 s ± 0.030) and after administration of levodopa [0.274 s ± 0.080; *t*(15) = 3.91, *P* = 0.001]. The significant relationship is also present when considering the mean of the individual median burst durations [0.297 s ± 0.014 versus 0.234 s ± 0.008; *t*(15) = 3.62, *P* = 0.003]. The percentage time of the total signal spent as bursts was equally distributed across hemispheres and conditions and higher during OFF levodopa compared to ON levodopa [27.0 ± 0.5% versus 16.1 ± 1.7%; *t*(15) = 4.95, *P* < 0.001] (Supplementary Fig. 3). The percentage burst time varied between hemispheres according to levodopa responsiveness. When considering the amplitude of beta bursts (Fig. 3B) we observed higher amplitude before administration of levodopa [0.176 arbitrary units (au) ± 0.039] compared to after administration of levodopa [0.122 au ± 0.023; *t*(15) = 2.856, *P* = 0.012].

Above we selected the 75th percentile threshold as our representative threshold to determine beta bursts. However, because of the arbitrary nature of threshold selection we also tested if the relationship between beta bursts OFF and ON levodopa is maintained using a range of different thresholds (55th to 90th percentile; Supplementary Fig. 4). This confirmed that mean burst duration decreased with rising amplitude threshold, while mean burst amplitude increased. Importantly, however, the difference between bursts OFF and ON levodopa was maintained across different thresholds, so that shorter bursts with lower amplitudes were systematically more common during ON levodopa compared to OFF levodopa.

Furthermore, we also contrasted burst duration and burst amplitude with the same burst parameters derived from a permutation of the raw LFP data using the same burst determination algorithm, but (as in corresponding Fig. 3) without prior categorization of burst durations into burst time windows (Supplementary Fig. 5). The results showed that in the OFF levodopa state the burst duration and burst amplitude were both higher compared to the parameters derived from the permutated data. In the ON levodopa state, burst duration of the shuffled data was similar to that of the original data, while burst amplitude was greater in the original dataset. These results suggest that, even ON levodopa, beta activity was organized into bursts of bigger amplitude than expected by chance given the nature of the LFP signal.

### Burst duration and burst amplitude are strongly related in both OFF and ON levodopa

**Figure 4:**
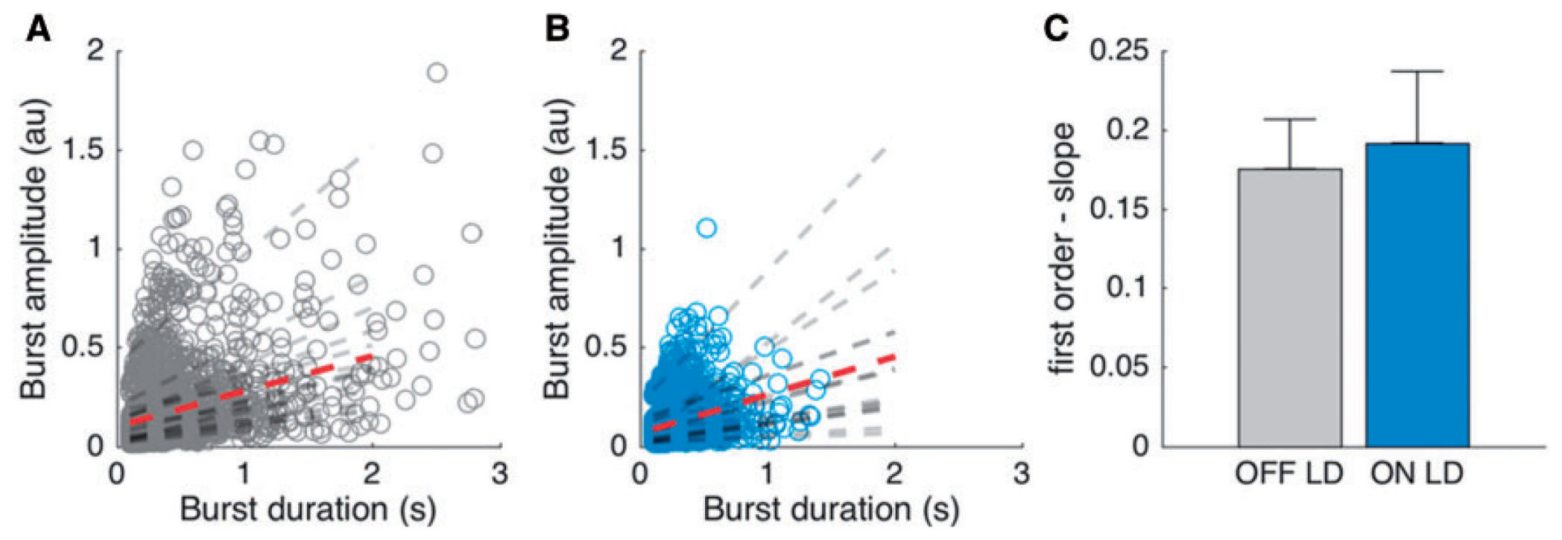
Relationship between burst duration and burst amplitude. (**A** and **B**) The relationship between burst duration (*x*-axis, depicted up to 3 s) and burst amplitude (*y*-axis) for all detected bursts across hemispheres. The dashed lines in grey show the first order fit between the two variables for each hemisphere in each condition. The dashed line in red shows the mean first order fits across all hemispheres. Each hemisphere shows a highly significant and positive correlation between burst duration and burst amplitude for both the conditions with a mean r-value of 0.814 ± 0.010 for OFF levodopa and 0.781 ± 0.013 for ON levodopa. The r-values do not differ between conditions [*t*(15) = −0.703, *P* = 0.493]. (**C**) Compares the slopes of the first order fits between OFF and ON levodopa, which are not significantly different from each other. Beta bursts were determined using the 75th percentile amplitude threshold. Values are represented as mean + SEM. LD = levodopa.

So far we have considered the duration and mean amplitude of beta bursts separately. But how are these related and how does levodopa impact on any such relationship? Figure 4 illustrates the relationship in the two conditions. Spearman correlation showed for each hemisphere a strong positive and highly significant correlation (OFF levodopa: mean Fisher-transformed r-value 1.15 ± 0.0325, *P* < 0.001 for all hemispheres; ON levodopa: mean Fisher-transformed r-value 1.06 ± 0.0340, *P* < 0.001 for all hemispheres). The gradient of these fits showed no difference between OFF and ON levodopa [*t*(15) = −0.703, *P* = 0.493], so that the differential distribution of burst durations between OFF and ON conditions largely determined the different mean burst amplitudes in the two states. However, Fig. 4 does not allow us to infer the shape of the beta bursts, only that the longer they lasted the higher the amplitude of the beta activity averaged across the duration of the burst. To address this, we measured where the peak of each burst fell as a percentage of the total burst duration. Regardless of drug state, the peak occurred around 50% into the evolution of the burst (OFF levodopa 50.0 ± 0.3%, ON levodopa 49.2 ± 0.3%), consistent with a spindle shape to the bursts.

### Bursts occur less frequently during ON levodopa and during movement

To investigate how often beta bursts occur, we calculated the burst frequency as bursts/s (Fig. 3C). We found that the frequency of bursts was lower during the ON levodopa (0.58 ± 0.056) compared to the OFF levodopa state [0.70 ± 0.03; *t*(15) = 3.52, *P* = 0.003], across all burst durations above 0.1 s. Supplementary Fig. 6 shows the distribution of the burst frequency for each time window before and after levodopa. This demonstrates that the reduction in burst frequency (Fig. 3C) is mainly driven by less frequently occurring long duration beta bursts during ON levodopa. Importantly, this comparison only takes into account beta bursts in both conditions, which have a sufficient magnitude to be detected by the common 75th percentile amplitude threshold and does not consider smaller variations of the time evolved wavelet amplitude. The probability of short bursts ON levodopa exceeded that OFF levodopa when lower common thresholds were used (Supplementary Fig. 7).

Four of the subjects (eight hemispheres) were also recorded while they made self-paced, discrete front-and-back joystick movements, as well as whilst they were seated quietly at rest. In this albeit small sample, beta burst frequency and duration were significantly attenuated when movements were made OFF levodopa, bringing beta burst characteristics more in line with those seen ON levodopa (Supplementary Fig. 8).

### Change in burst properties is preserved if definition of beta bursts is changed

As part of the signal processing to derive beta bursts we selected the wavelet transformed time signal corresponding to the beta peak frequency (1 Hz bin, see Table 1). This raises the possibility that, rather than a genuine shift in beta bursts from long to short duration during ON levodopa, a shift in burst frequency may lead to an over or underestimation of burst duration at the original peak frequency. To mitigate against this possibility, we repeated the signal processing using a much broader definition of the beta peak (beta peak ± 5 Hz) (Supplementary Fig. 9). The difference in burst duration was preserved between OFF (0.39 s ± 0.018) and ON (0.31 s ± 0.01) medication [*t*(15) = 3.944, *P* = 0.001]. The same was true for burst amplitude, which was higher during OFF compared to ON [*t*(15) = 3.14, *P* = 0.007] drug. These data suggest that changes in burst duration are not a consequence of a shift in beta peak frequency.

Similarly, both burst duration and amplitude were reduced during ON levodopa compared to OFF levodopa when the individual 75th percentile amplitude threshold in each state was used to derive beta bursts instead of the common amplitude threshold averaged across drug states (Supplementary Fig. 10). This suggests that key state differences were not simply the product of differently scaled signals, but rather of real changes in the relative distribution of burst durations and amplitudes within the two drug states. Moreover, the findings were also preserved if we considered the variation of the entire time evolved wavelet amplitude of the peak beta activity, without being restricted to periods above a certain threshold. Supplementary Fig. 11 illustrates the relative variability of the time-evolved wavelet amplitude (that was also selected for burst determination) for both the conditions. The relative amount of slower amplitude variation (<1 Hz) that corresponds to longer burst durations is greater during the OFF levodopa condition, while the faster amplitude variation (>1 Hz), which corresponds to shorter burst durations, is relatively greater during the ON levodopa condition.

### Beta bursts are coupled between hemispheres

**Figure 5:**
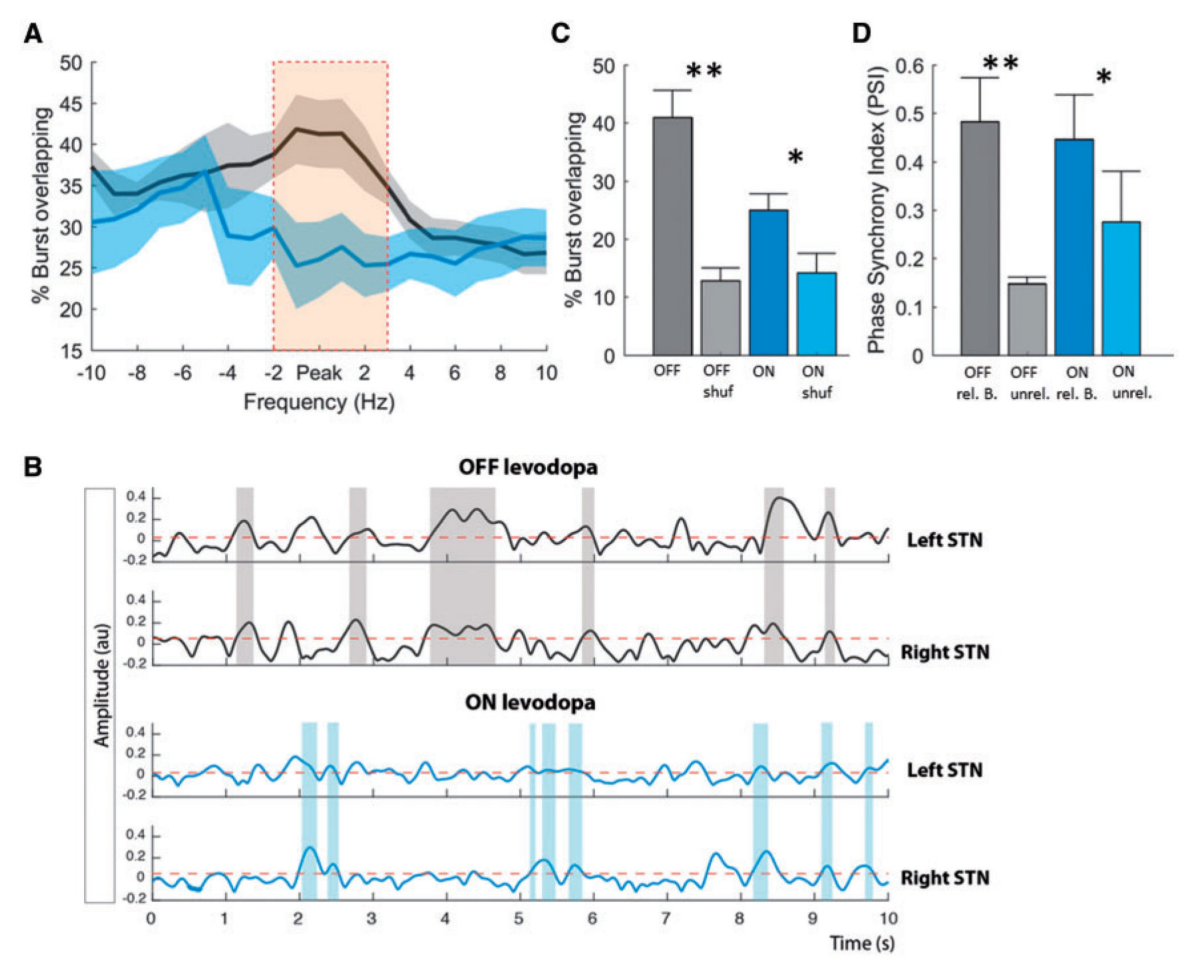
Burst coupling between hemispheres. (**A**) The percentage of the total number of bursts that overlap in time between hemispheres for the peak frequency of beta (averaged across the two sides) and surrounding frequencies. Data were realigned to the frequency of the beta peak in each STN and then averaged. During OFF levodopa the percentage burst overlapping is significantly higher compared to ON levodopa and also frequency-specific around the beta peak (cluster-based permutation test significance shown by orange bar). (**B**) Illustrates 10 s of simultaneous time evolving wavelet amplitude for the beta peak frequency for the left and right hemisphere and both OFF (grey) and ON (blue) levodopa. This illustrates the stronger burst overlapping (shaded areas) during OFF levodopa compared to ON levodopa (Subject 7). (**C**) Contrasts the difference in percentage overlapping between the conditions for the beta peak frequency, with the overlapping by chance (shuf = shuffled data). Both the conditions show a stronger overlapping compared to that expected by chance, with no difference in the overlapping by chance between the conditions. (**D**) The PSI between hemispheres for related-overlapping and shuffled unrelated-overlapping burst (B = bursts) periods OFF and ON levodopa. The PSI for related bursts is much higher compared to unrelated bursts for both the conditions. Beta bursts were determined using the 75th percentile amplitude threshold. Values are represented as mean + SEM; *P < 0.05.

Hitherto we have examined the properties of beta bursts within one hemisphere. How are beta bursts related between hemispheres? To investigate the inter-hemispheric relationship between beta bursts, we first calculated the percentage of burst periods that overlapped between the left and right STN (Fig. 5A). We considered the beta peak frequency and the neighbouring 10 1-Hz bins above and below this. The difference in the percentage overlapping was frequency-specific and greatest around the individual beta peak. Figure 5B illustrates the interhemispheric burst overlapping on a 10 s signal period for both OFF and ON levodopa. Bursts at the beta peak frequency overlapped OFF levodopa by 40.93 ± 4.71%, and ON levodopa by 24.98 ± 2.82% (Fig. 5C). Furthermore, for both the conditions beta bursts were greater in their overlap than might be expected by chance [*t*(15) = 4.71, *P* = 0.002; *t*(15) = 2.64 *P* = 0.03] (Fig. 5C). Importantly, even though the duration of beta bursts was similar between real data and shuffled data ON medication, there was more overlapping of beta bursts between the two hemispheres in the original data than the shuffled data. This indicates that the increased overlapping could not just be due to longer bursts in the two hemispheres. Next, we calculated the PSI for related-overlapping bursts and shuffled unrelated-overlapping bursts. We found a significantly stronger PSI for related compared to unrelated beta bursts for both OFF [*t*(15) = 3.60, *P* = 0.009] and ON levodopa [*t*(15) = 2.69, *P* = 0.0313] (Fig. 5D). However, there was no significant difference in PSI between bursts OFF and ON levodopa if cumulative burst durations were matched and compared between conditions [*t*(15) = 0.9011, *P* = 0.398]. These results suggest that the phase difference between the two hemispheres in the beta band was consistent across all bursting periods for both OFF and ON levodopa.

### Clinical correlation

**Figure 6:**
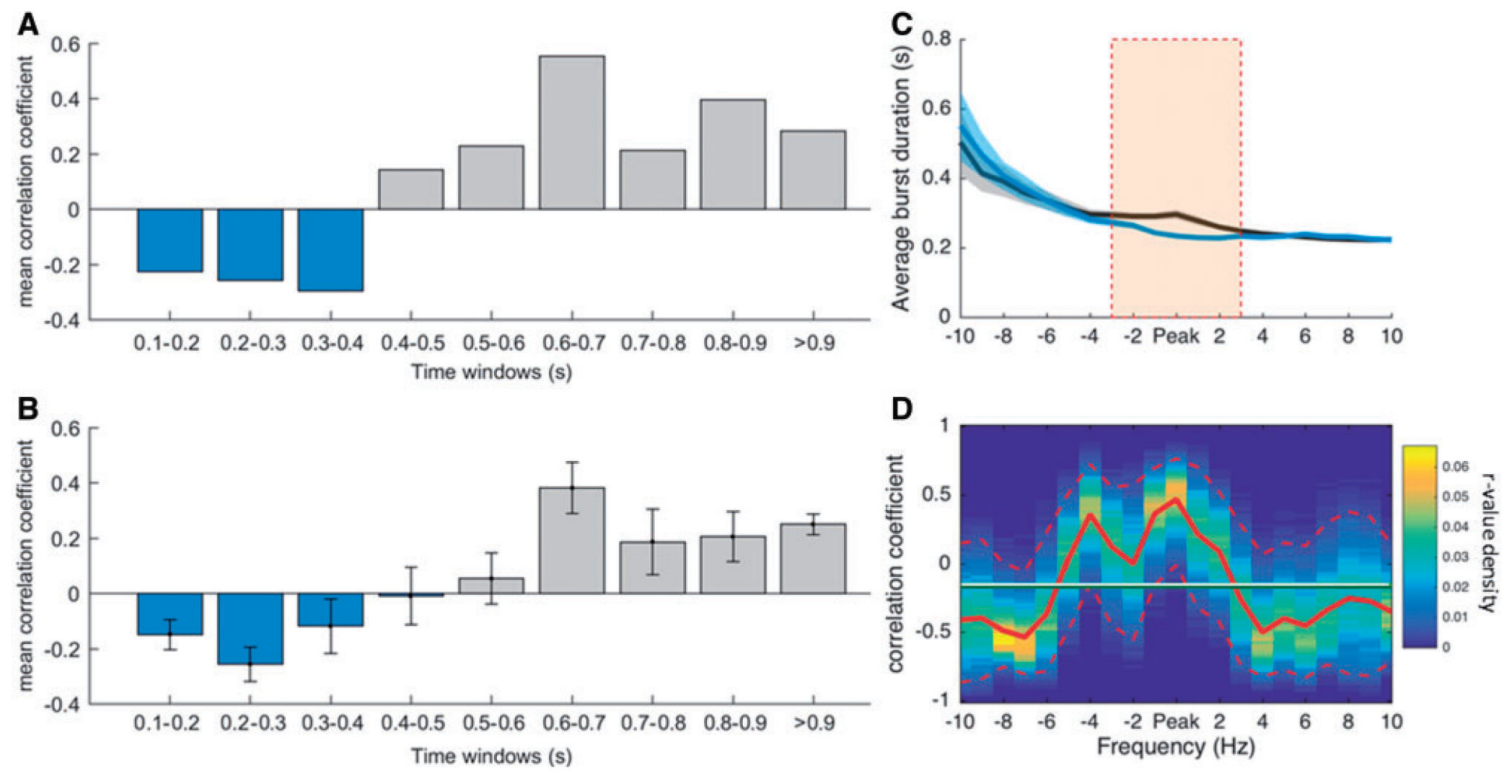
Clinical correlation. (**A**) Fisher transformed Spearman’s correlations between clinical impairment (total UPDRS items 20–26) and the percentage amount of bursts during bursts of different durations for the OFF levodopa condition and 75th percentile amplitude threshold. These show that a higher amount of shorter bursts tend to be negatively correlated with clinical impairment and a higher amount of longer bursts tend to be positively correlated with clinical impairment. (**B**) Shows the same analysis as in **A** with Fisher transformed r-values averaged across various percentile amplitude thresholds (55–90 percentile). The pattern of shorter bursts being negatively correlated with clinical impairment and longer bursts being positively correlated, is not specific for the 75th percentile thresholds, but consistent across different thresholds. (**C**) Illustrates the average of the median burst durations for OFF and ON levodopa, for the peak frequency of beta and 10 neighbouring frequencies across sides. The significant changes in burst duration are frequency-specific and located around the beta peak frequency (cluster-based permutation test significance shown by orange bar). (**D**) The r-values of the correlation between the ratio of median burst duration between the conditions and the motor improvement in contralateral hemibody UPDRS at the beta peak frequency ± 10 Hz frequency bins. The positive correlation is highest at the individual beta peak frequency. The horizontal line illustrates the mean r-value, the red dashed lines show the 95 confidence limits of the r-value density distribution of 10 000 bootstrap cycles (bootstrap method). Values are represented as mean ± SEM (bars and shaded areas). The relationships for burst duration and UPDRS sub-items are illustrated in Supplementary Fig. 12 and 13.

Finally, to investigate how burst duration in the OFF levodopa state was related to clinical impairment, we correlated the percentage number of beta bursts in a given interval within different burst duration time windows with clinical impairment (Fig. 6A), repeating this across different thresholds (Fig. 6B). This showed that the percentage number of beta bursts of longer duration was positively correlated with clinical impairment, while the opposite was true for bursts of shorter duration. When considering the UPDRS sub-items (bradykinesia, rigidity and tremor) we found a similar relationship, which however was weaker for the tremor sub-item (Supplementary Fig. 12).

We also investigated how the relative change in burst duration impacted on the clinical performance after administration of levodopa. Figure 6C illustrates the median burst duration for both the conditions over a range of frequencies (beta peak ± 10 Hz). Differences were significant around the individual beta peak (cluster-based permutation test). The ratio between burst duration OFF and ON levodopa was then correlated with the clinical improvement after levodopa administration (Fig. 6D). Spearman’s rho between decrease in burst duration and clinical improvement was highest around the individual beta peak frequency. Again, similar trends were observed when UPDRS sub-items (bradykinesia, rigidity and tremor) were considered individually (Supplementary Fig. 13). The 95th confidence interval around the beta peak was above the mean r-value across frequency bins and the beta peak area (Fig. 6C, peak ± 3 Hz) was significantly different from the non-peak area [*t*(15) = 4.0884, *P* < 0.001]. In summary, the change in the relative distribution of burst durations between ON and OFF medication was linked to the change in clinical state. Supplementary Fig. 6 suggests that precisely what drove this change in distribution depended on the common amplitude threshold used: with higher amplitude thresholds the reduction in long duration bursts by levodopa dominated, whereas with lower amplitude thresholds there was an additional shift in favour of more short duration bursts ON levodopa (Supplementary Fig. 7).

## Discussion

Our findings demonstrate that levodopa treatment changes the relative distribution of beta bursts in the subthalamic nucleus from long to short duration in patients with Parkinson’s disease withdrawn from drug treatment, so that there are more long duration bursts OFF compared to ON levodopa. The importance of this is that beta burst amplitude progressively increases with burst duration. The increase in burst amplitude as bursts last longer is indicative of increasing local synchronization within the beta band, and elsewhere we have speculated that excessive synchronization at the local and circuit level can compromise information coding capacity and thereby motor processing (Brittain and Brown, 2014). In line with the presence of more distributed synchronization, we found that beta bursts are much more likely to occur simultaneously and to be phase coupled across hemispheres than by chance in Parkinson’s disease patients. Clinical correlations are consistent with a deleterious effect of hypersynchronization in long duration beta bursts. The percentage number of longer beta bursts in a given interval OFF levodopa is positively correlated with clinical impairment (with the opposite true for the percentage number of shorter beta bursts). Importantly, the decrease in burst duration after administration of levodopa is also correlated with improvement in motor deficit.

In redistributing beta bursts in favour of those of shorter duration and smaller amplitude, levodopa therefore has similar effects to adaptive DBS. The same study showed that the percentage number of short and long bursts differentially correlate with motor impairment (Tinkhauser *et al.*, 2017). The parallels between the effects of dopaminergic therapy and those of adaptive DBS help motivate the development of the latter and to focus attention on the dynamics of beta bursts as a rational target for closed-loop DBS.

### The likelihood and duration of beta bursts

The levodopa-driven change in the relative distribution of longer and shorter beta bursts was striking, could be replicated using a selection of different burst amplitude thresholds, and was also evident in the spectral domain. The latter confirmed a relative shift in favour of amplitude variability of higher frequency following levodopa administration indicative of a relative reduction in burst duration, despite this being derived with an amplitude independent method and thus not limited to suprathreshold signal periods. Importantly, we were not just thresholding signals with different standard deviations (differences in means were removed by DC correction in our signal processing pipeline) between drug states. Those bursts defined by our standard, common 75% amplitude threshold were longer in the OFF levodopa state and bigger in amplitude in both drug states than expected by chance. Other evidence that bursts were not the product of simply thresholding signal variance was their rich overlapping across hemispheres and the bilateral synchronization during these overlapping bursts. Moreover, differences in burst duration and amplitude between drug states were preserved when we used condition-specific amplitude thresholds. Finally, the similarity of the results when using a broad as opposed to a narrow beta frequency bandwidth militated against a potential shift in beta peak frequency as an explanation for the change in burst duration (Tinkhauser *et al.*, 2017).

The shift in the relative distribution of beta bursts towards shorter durations following treatment with the dopamine prodrug levodopa raises the possibility that such bursts are more in keeping with the physiological state. Such a conclusion is supported by studies conducted in healthy non-human primates, which describe beta bursts in the motor network as rather short events, lasting for a few cycles only (Murthy and Fetz, 1992, 1996; Feingold *et al.*, 2015). Also consistent with the hypothesis that fewer and shorter bursts my be closer to the physiological state is the fact that successful joystick movements made OFF levodopa were accompanied by a temporary reduction in the frequency and duration of beta bursts.

In addition, beta bursts tended to be not only of shorter duration and smaller amplitude on levodopa but were also reduced in their probability, at least when using a reasonably conservative amplitude threshold. Previous work conducted in healthy non-human primates suggests that the diminution of burst probability may underpin movement-related beta de-synchronization in the striatum (Feingold *et al.*, 2015), and highlights burst likelihood as being another functionally relevant parameter. Together, reduced burst duration and hence amplitude, and reduced burst probability following treatment with levodopa will contribute to the widely reported suppression of mean beta levels in the ON drug state (for review see Hammond *et al.*, 2007). It remains to be seen, however, whether these differences are sufficient to explain all of the suppression in mean beta levels.

### Relationship between burst duration and synchronization

Why might the duration of beta bursts matter? For beta activity to be recorded in the LFP neuronal activity has to be synchronized so that spatiotemporal summation occurs. In the STN such synchronization is likely to be mainly due to afferent, synaptic activity (Weinberger and Dostrovsky, 2011). As the duration of beta bursts increases so does the amplitude, indicative of progressive synchronization of inputs over time. A similar relationship between burst duration and degree of synchronization has also been reported in striatal recordings of non-human primates suggesting it might be a general phenomenon of circuit dynamics where some degree of lateral connectivity is present at the input level or through intrinsic connectivity (Feingold *et al.*, 2015). Indeed, the phenomenon of increasing synchronization over time as well as the coupling of co-occurring beta bursts between hemispheres may be an emergent property of neurons acting as weakly coupled oscillators through network resonance (Hahn *et al.*, 2014). These effects in patients were present for beta bursts both OFF and ON levodopa without difference in the gradient relating duration to amplitude, shape of bursts or in interhemispheric PSI, leading us to posit that the difference between the OFF and ON drug states may partly lie in the timing of the termination of synchronization, i.e. of burst duration (Park *et al.*, 2010; Cagnan *et al.*, 2015). Thus, a similar therapeutic effect can be achieved by artificially limiting burst duration with adaptive DBS (Tinkhauser *et al.*, 2017). Significantly, this last observation also provides evidence that the correlation between long duration bursts and motor impairment may arise because long duration, higher amplitude beta bursts are causally important in determining motor impairment.

Strikingly, it was not just local synchronization that was evident in beta bursts. Periods of high amplitude beta substantially overlapped across hemispheres; less so ON than OFF levodopa but still more than expected by chance. Moreover, the phase synchrony during cotemporaneous bilateral bursts was greater than between shuffled-not related beta bursts in the same subject, but was not different between drug states. Previous studies have shown that beta activity is coherent between STNs (de Solages *et al.*, 2010) and that this coherence is disrupted after administration of levodopa (Little *et al.*, 2013*b*), but they have not considered the dynamic nature of synchronization. Our findings suggest that the disruption of coherence between STN activities by levodopa may be predominantly caused by a reduction in burst probability and abbreviation of synchronized bursts in the two hemispheres.

### Study limitations

Importantly, recordings and clinical testing took place in the immediate postoperative phase and a confounding stun effect cannot be excluded (Chen *et al.*, 2006). The effect of levodopa on beta bursts should therefore be confirmed in chronically implanted patients. We have also distinguished between physiological, short duration, low amplitude beta bursts and pathological, longer duration, higher amplitude bursts, whereas it is likely that the evolution from physiological to pathological Parkinson’s disease-related beta bursts is a continuum, without a clear demarcation that allows for a dichotomized categorization of beta bursts. Rather, it may be the relative distribution of bursts in terms of duration and amplitude that better serves to characterize the normal and disease state, and we should acknowledge here the possibility that the precise burst distribution characterizing the physiological state may not be fixed but vary according to context and task. In this regard, it is important to note that we found that the probability and duration of beta bursts were diminished during movement, at least when OFF levodopa. Finally, in our analysis we focused on the dynamics of beta activity, and it remains possible that background tonic levels of beta activity are also relevant in determining clinical state.

### Implications for adaptive DBS

The transition from negative to positive correlation between burst duration and motor impairment occurred with burst durations of ~400–500 ms, assuming a threshold burst amplitude of 75%. This provides a benchmark for adaptive DBS, which therefore would be best delivered so as to trim bursts of longer duration than this and, equally, to leave unaffected those that are shorter than this. Through good fortune, rather than design, the adaptive DBS regimes that have been shown to be as, or more, effective as conventional continuous DBS in patients OFF medication have only been able to kick-in when burst durations exceeded a similar critical duration (Little *et al.*, 2013*a*, 2016*a*, *b*; Pina-Fuentes *et al.*, 2017). Our findings raise the possibility that adaptive systems that aim to shorten the duration of beta bursts with a bang-bang (on/off regulation) control algorithm may be preferable to those involving a more gradual proportional–integral–derivative control policy with substantial signal smoothing. Depending on the degree of smoothing involved, the latter may miss burst events or affect short duration bursts. Nevertheless, further studies are required to determine the most efficacious closed loop control algorithm and then to compare the clinical performance of the adaptive DBS system with the optimized control algorithm with that provided by established conventional DBS.

Our results also raise the possibility that stimulation may need to be triggered even less often in the case of combined levodopa treatment and adaptive DBS. This is because of the reduction in burst probability and duration after administration of levodopa. In the case of STN DBS this could automatically prevent dyskinesia by reducing the sum effect of stimulation and medication. Indeed in a previous adaptive DBS clinical trial it was seen that with increasing levodopa effect, stimulation was triggered less often (Little *et al.*, 2016*a*) and other trials have shown that dyskinesia are suppressed during adaptive DBS compared to conventional DBS (Rosa *et al.*, 2015, 2017).

## Conclusion

**Figure 7:**
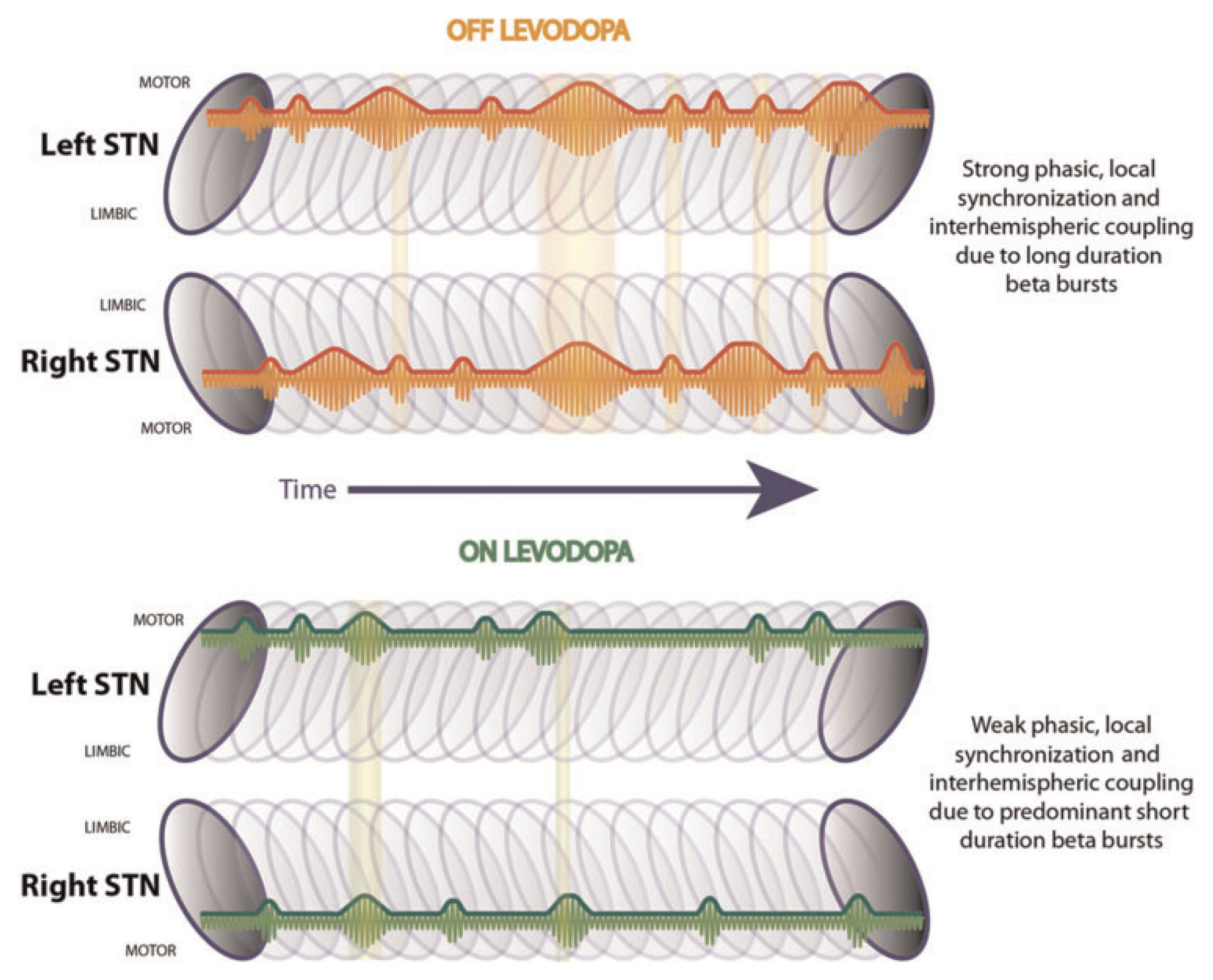
Summary schematic. Illustrates the left and right STN with a series of beta bursts during OFF and ON levodopa located in the dorso-lateral motor region (Horn *et al.*, 2017). During OFF levodopa there are short and long duration beta bursts, while during ON levodopa shorter bursts are predominant. Long duration beta bursts lead to a stronger phasic synchronization within the STN motor region, which is related to motor impairment in Parkinson’s disease. Beta bursts co-occur between hemispheres and are phase coupled, while these overlapping periods are more common during OFF levodopa compared to during ON levodopa.

Here we provide evidence that pathological beta activity consists of prolonged bursts in Parkinson’s disease, that these bursts are associated with excessive synchronization within bilateral basal ganglia circuits and that longer duration bursts are abbreviated and made less frequent by treatment with the dopaminergic prodrug, levodopa (Fig. 7). Increases in the relative numbers of longer beta bursts are correlated with clinical impairment, whereas the reduction in burst duration correlates with improvement in motor deficit. These observations provide correlative evidence that the distribution of burst durations distinguishes the parkinsonian (OFF) and more physiological (ON) state and may help determine motor function or deficit. Adaptive DBS may mimic the effect of levodopa in biasing burst dynamics in favour of relatively shorter, smaller bursts.

## Supplementary Material

Supplementary material

## Figures and Tables

**Table 1:** Clinical details

Subject	Sex	Age, years	Disease duration	Dominant symptoms	Total UPDRS OFF/ON levodopa (mg)	Stimulation site	Beta peak frequency (Hz)
1	F	62	12	Bradykinesia, dyskinesia	37/16.5 (100 mg)	L R	25 24
2	M	69	18	Bradykinesia	52.5/29.5 (200 mg)	L R	29 17
3	F	48	8	Bradykinesia, dyskinesia, tremor	21.5/4 (200 mg)	L R	18 18
4	M	69	11	Bradykinesia, dyskinesia, freezing	24/18 (250 mg)	L R	14 16
5	M	57	17	Tremor	29.5/18 (200 mg)	L R	18 19
6	M	65	14	Tremor, motor fluctuation	38/28.5 (200 mg)	L R	25 25
7	F	63	5	Tremor	14.5/11 (200 mg)	L R	17 29
8	M	67	16	Tremor	46.5/25.5 (200 mg)	L R	12 12
Mean ± SEM	F(3): M(5)	62.5 ± 2.5	12.6 ± 1.6		33.0 ± 4.6/18.9 ± 3.1 (193.8 ± 14.8)		19.9 ± 2.0

F = female; L = left; m = male; OFF/ON levodopa represents the preoperative motor scores before and after the bracketed test dose of levodopa; R = right.

## Abbreviations

DBS: deep brain stimulation
LFP: local field potentials
PSI: phase synchrony index
STN: subthalamic nucleus
UPDRS: Unified Parkinson’s Disease Rating Scale
