# Supplementary material for: Beta burst dynamics in Parkinson’s disease OFF and ON dopaminergic medication
Source: Brain. Author manuscript; Available in PMC 2017 Nov 2. (PMC5667742; doi:10.1093/brain/awx252)
Supplement: Supplementary material [file NIHMS74682-supplement-Supplementary_material.pdf]

Supplementary material

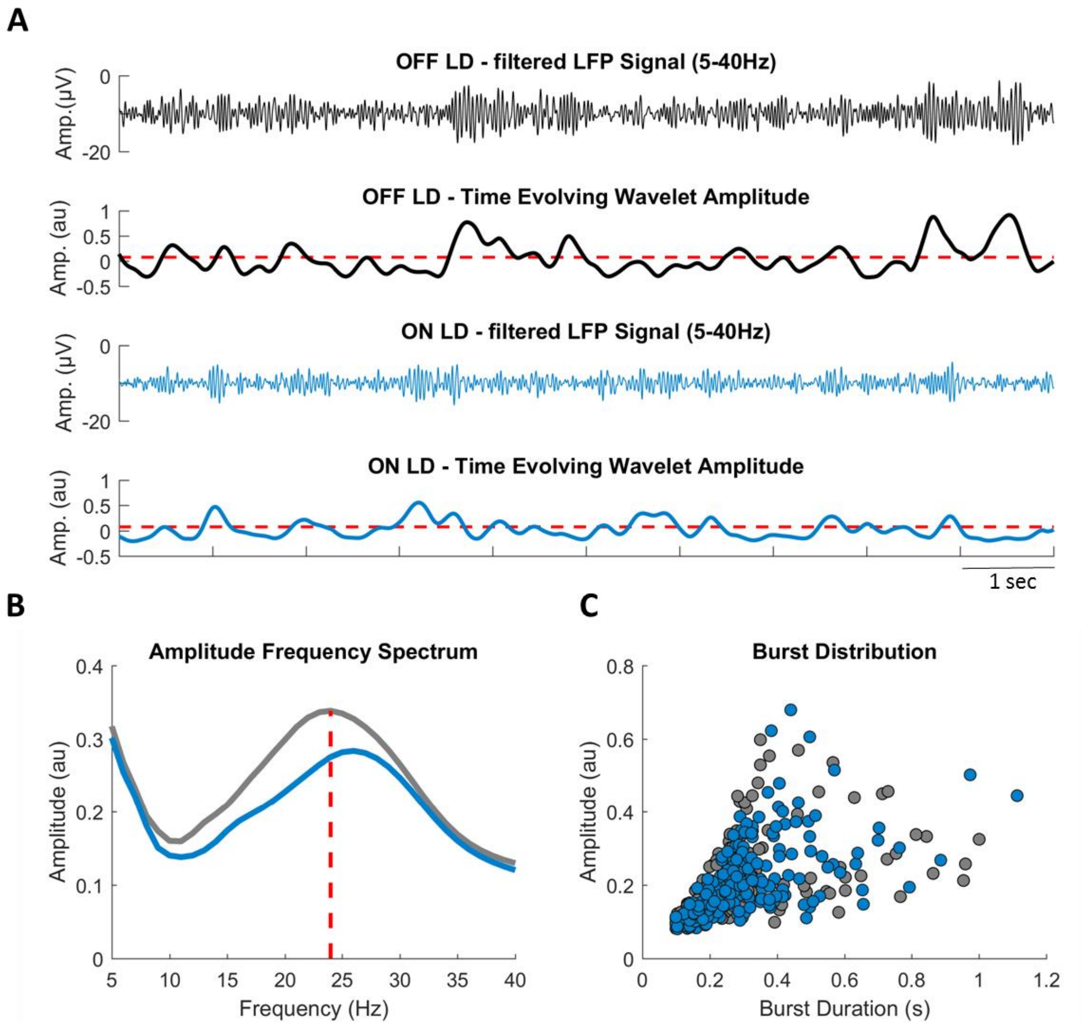

Supplementary figure 1: Burst determination (A) illustrates a segment of the filtered LFP signal (5-40Hz) and the time evolving wavelet amplitude (from the same segment) of the beta peak frequency (24 Hz) derived from the wavelet transformed signal, both for OFF (grey) and ON (blue) levodopa. The red dashed horizontal line illustrates the common amplitude threshold, which corresponds to the mean of the 75th percentile amplitudes of OFF and ON levodopa. Periods of the time evolving wavelet amplitude that cross this threshold for longer than 0.1 seconds were defined as beta bursts. **(B)** shows LFP amplitude spectra for OFF and ON levodopa, with a beta peak at 24Hz in the OFF levodopa condition and reduction of beta amplitude in the ON levodopa condition. **(C)** shows amplitude and duration of all detected beta bursts for both the conditions taken from recordings of 201s and 319s duration. Example subject 1, right side.

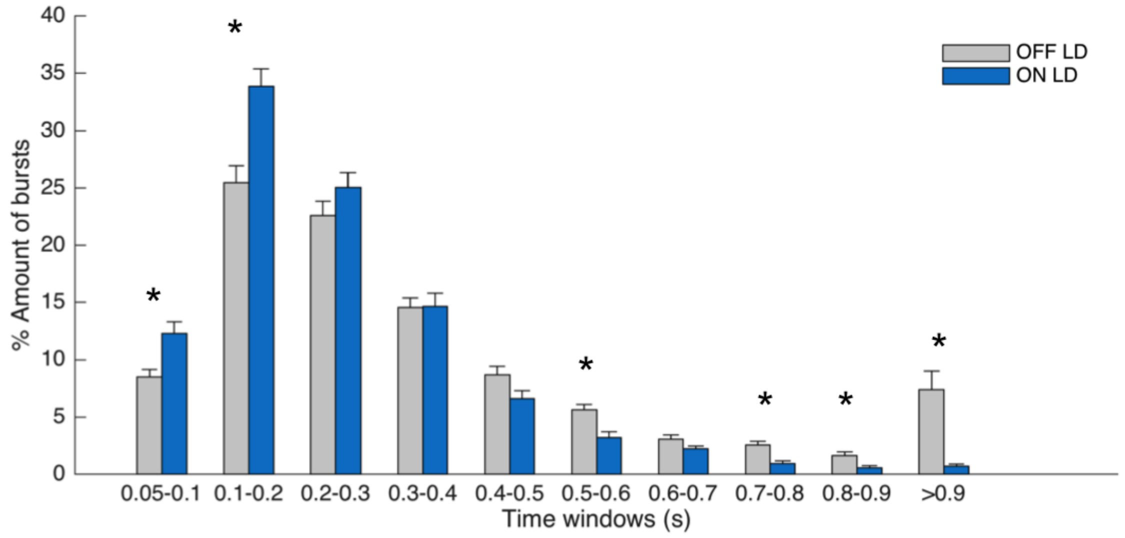

Supplementary figure 2: Change in burst duration distribution including very short bursts <0.1s. Distribution of burst durations averaged across 16 sides as a percentage of total number of bursts on each side, during OFF levodopa and ON levodopa, where bursts are defined as periods of beta activity that exceed the 75th percentile amplitude threshold with a minimum duration of 0.05s. A RM-ANOVA showed a significant main effect for the interaction between condition and burst duration ($F_{(df\ 2.420)}$=11.672, p<0.001). The corresponding post hoc comparison between OFF levodopa and ON levodopa showed that the percentage number of shorter beta bursts (0.05-0.1s; 0.1-0.2s) over a given interval was higher during ON levodopa compared to OFF levodopa (t_{15}=-4.607, p=0.001; t_{15}=-3.873 p=0.003). In contrast, the percentage number of longer bursts (0.5s-0.6s; 0.7s-0.8s; 0.8s-0.9s; >0.9s) was higher during OFF levodopa compared to ON levodopa (t_{15}=4.511, p=0.001; t_{15}=5.046, p=0.001; t_{15}=2.405, p=0.049; t_{15}=3.926, p=0.003). Values are represented as mean + SEM; *p_{corr}<0.05.

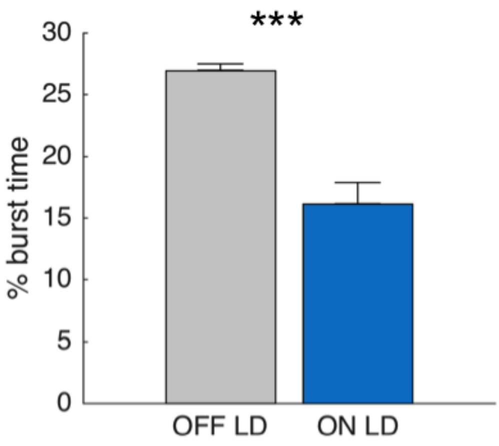

Supplementary figure 3: % time of the total signal spent as bursts during OFF and ON levodopa. This illustrates the mean percentage burst time, calculated for the common 75th percentile threshold, for both OFF and ON levodopa. The percentage burst time during OFF levodopa was on average 27.0 ± 0.5 % (range: 23.5% to 30.8%) and significantly higher (t_{15}=4.95, p<0.001) compared to the percentage burst time during ON levodopa which was on average 16.1 ± 1.7% (range: 3.6% to 24.0%). The reduction in the percentage burst time during ON levodopa could be explained in part by the reduction of the number of long duration beta bursts as well as by the reduced burst probability during ON levodopa. Although the percentage burst time varied with the individual levodopa response, values were equally distributed across hemisphere and conditions. Values are represented as mean + SEM; ***p<0.001.

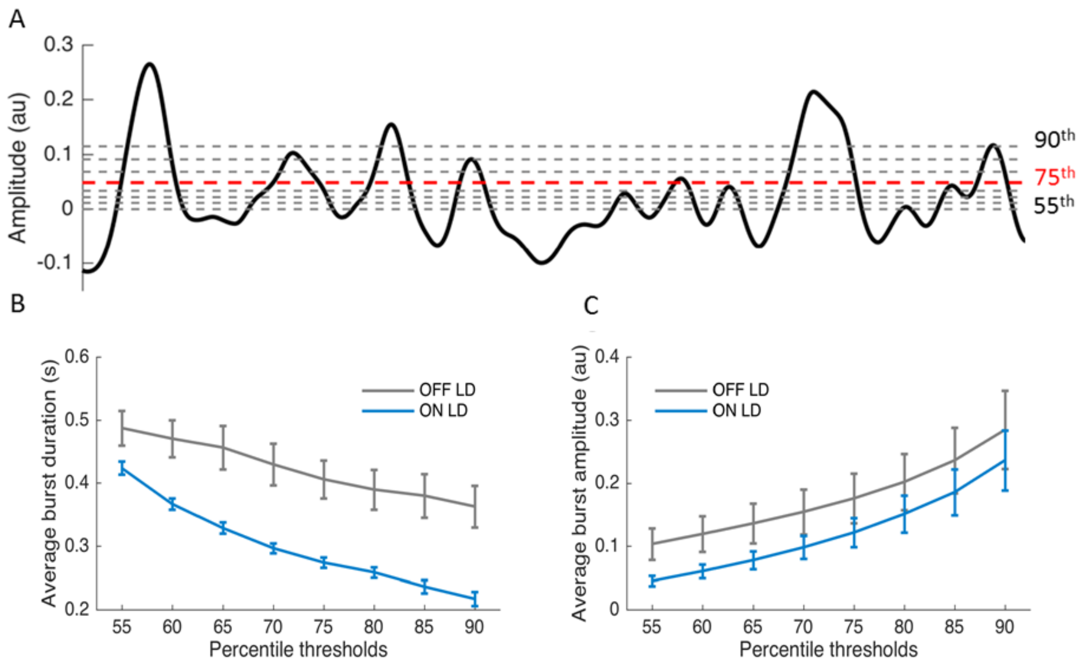

Supplementary figure 4: Burst amplitude and burst duration across different percentile amplitude thresholds (55-90). (A) example of an amplitude envelope with 8 different thresholds superimposed (55-90 percentile). The representative 75th percentile threshold is depicted in red. The mean burst duration **(B)** and the mean burst amplitude **(C)** are illustrated for both conditions across the different thresholds. With increasing percentile threshold the burst duration decreases, while the burst amplitude increases in both conditions. Importantly, the relationship between bursts OFF and ON levodopa is maintained across different thresholds, so that shorter bursts with lower amplitudes are more common during ON levodopa compared to OFF levodopa. Values are represented as mean ± SEM.

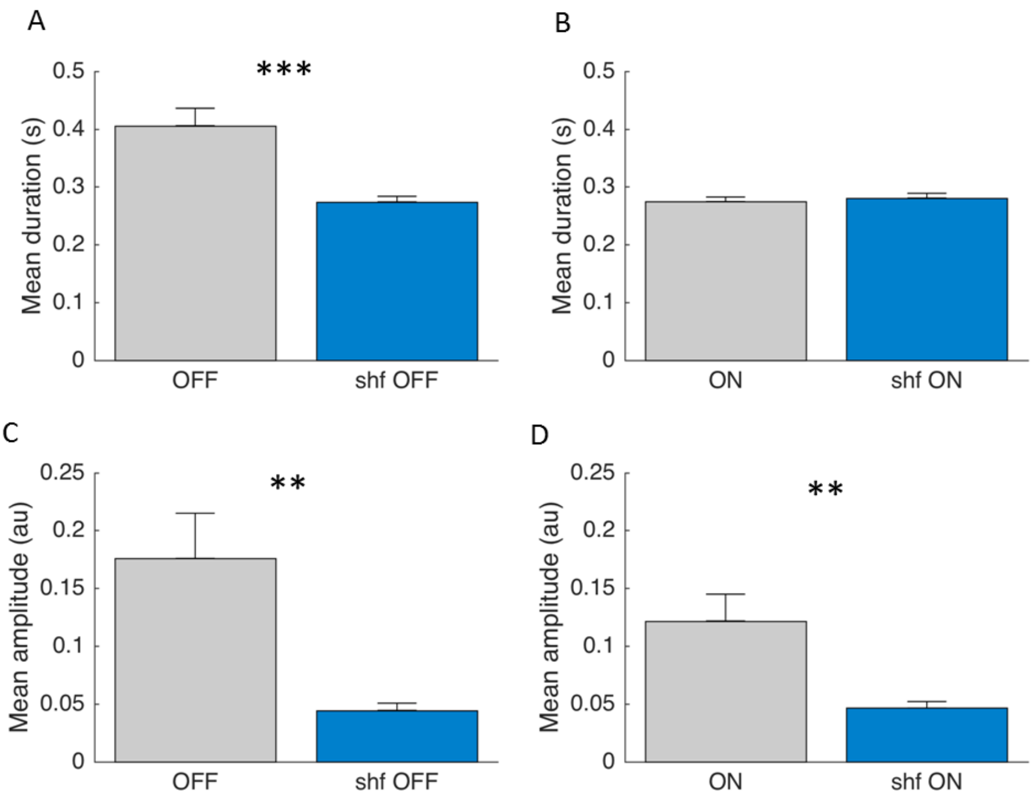

Supplementary figure 5: Burst duration and burst amplitude for shuffled data. This illustrates the mean burst duration (derived from the individual mean burst duration, without prior categorisation into burst time windows) and burst amplitude after application of the same burst determination algorithm on shuffled raw data (raw LFP signal permuted 1000 times) for both the conditions. **(A)** and **(B)** show the paired comparison between mean burst duration for unshuffled and shuffled (shf) data OFF (a) and and ON (b) levodopa. **(C)** and **(D)** show the paired comparison between burst amplitude for unshuffled and shuffled (shf) data for OFF (c) and ON (d) levodopa. The mean burst duration and amplitude in the OFF condition is higher in the original data compared to that seen for the shuffled data. For the ON condition the duration of bursts is similar to that seen for the shuffled data, however the burst amplitude is still higher, which confirms that beta bursts are still more structured in the ON levodopa state compared to shuffled data. Beta bursts were determined using the 75th percentile amplitude threshold. Values are represented as mean + SEM; *p<0.05, **p<0.01.

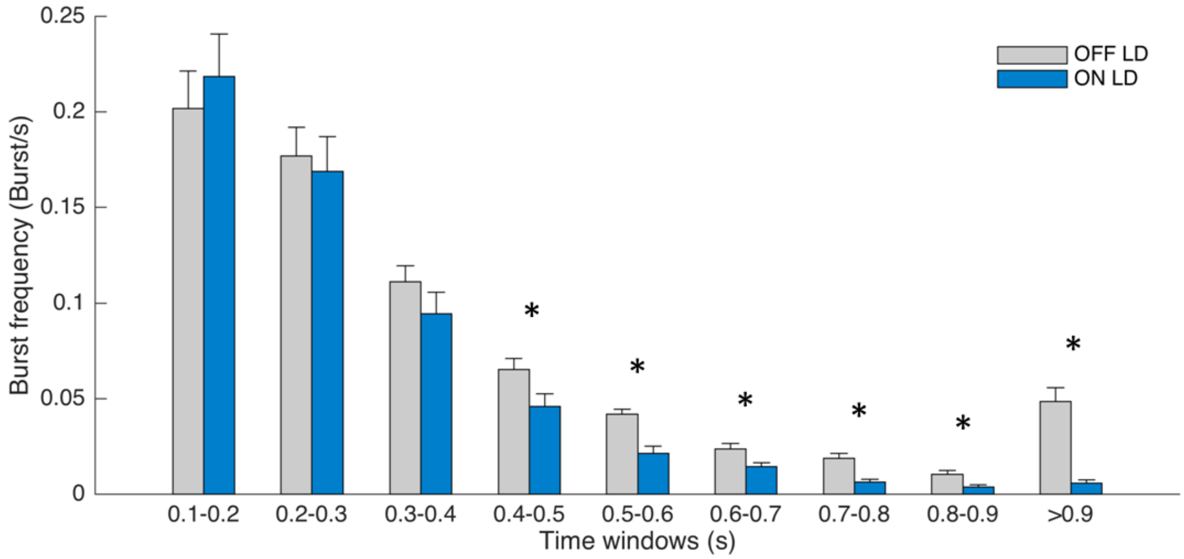

Supplementary figure 6: Burst frequency within different burst time windows (common 75th percentile amplitude threshold used). Shows the distribution of the burst frequency (bursts/s) for each time window before and after levodopa. The RM-ANOVA shows a significant main effect for the interaction between burst frequency and time windows ($F_{(df\ 3.639)}$=5.284, p=0.004). This demonstrates that the reduction in burst frequency (figure 3C) is mainly driven by less frequently occurring long duration beta bursts (> 0.4s) during ON levodopa. Values are represented as mean + SEM; *p_{corr}<0.05.

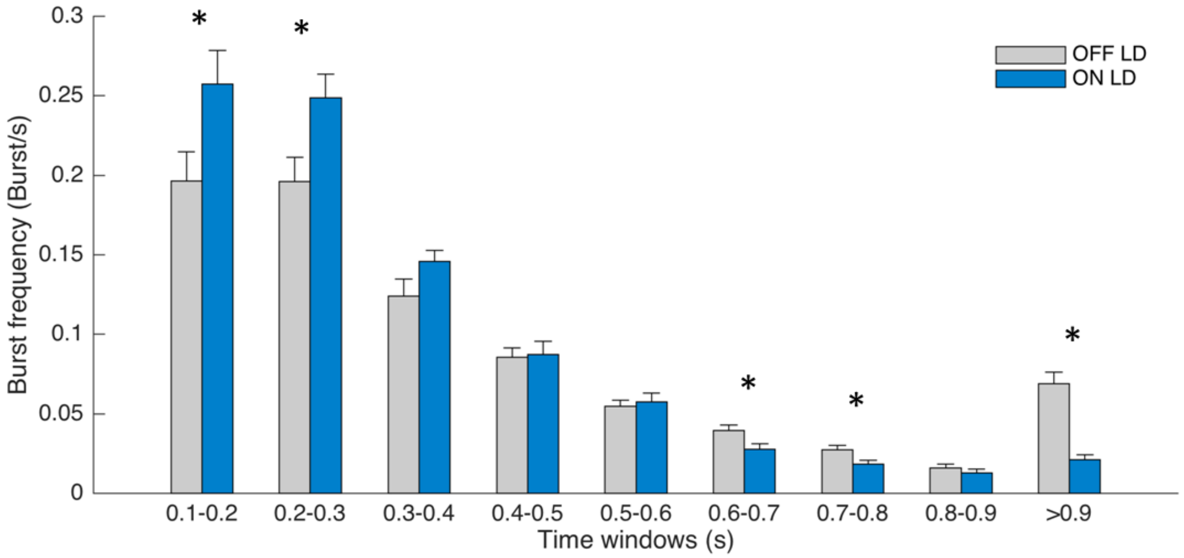

Supplementary figure 7: Burst frequency within different burst time windows (common 65th percentile amplitude threshold used). Shows the distribution of the burst frequency for each time window before and after levodopa. The RM-ANOVA shows a significant main effect for the interaction between burst frequency (bursts/s) and time windows ($F_{(df\ 3.205)}$=14.667, p<0.001). This demonstrates that the reduction in burst frequency (figure 3C) is mainly driven by less frequently occurring long duration beta bursts (> 0.5s) during ON levodopa, but that this was partially offset by a rise in the frequency of short bursts (0.1-0.2s and 0.2-0.3s duration) ON levodopa. Values are represented as mean + SEM; $^{*}p_{corr}<0.05$.

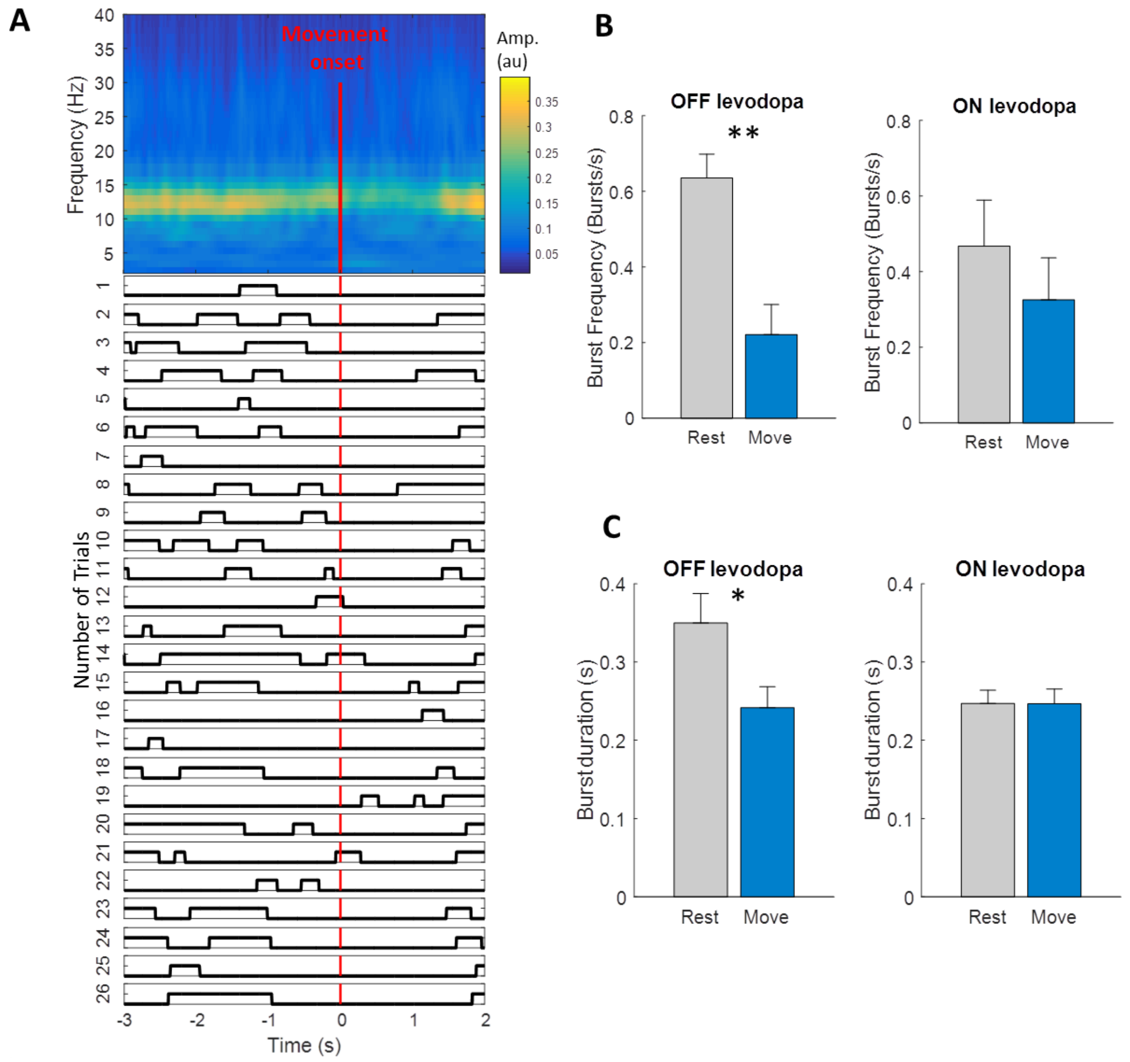

Supplementary figure 8: Beta bursts during movement related beta desynchronization. A: illustrates a time frequency spectrum averaged across trials of discrete self-paced joystick movements, with below the corresponding single trials plotted with beta bursts as binary events (burst or no burst present at any given moment). The averaged power spectrum indicates that beta desynchronization lasts about 1 second after movement onset and single trials indicate that during this period beta bursts occur less frequently. **B** and **C** show mean burst frequency (bursts/sec) and burst duration before and after movement onset for both OFF and ON levodopa. OFF levodopa there is a significant reduction in burst frequency during movement (0.22 ± 0.08 bursts/s) compared to the period before movement onset (0.64 ± 0.06) (z=-2.521, p=0.012; 8 pairs: neg. ranks=8, pos. ranks=1). During OFF levodopa the burst duration during movement (0.24 ± 0.03s) was significantly shorter compared to the period before movement (0.35 ± 0.04s) (z=-2.028, p=0.043; 7 pairs: neg. ranks=6, pos. ranks=1). In the ON levodopa condition there is a trend for a reduced burst frequency during movement (0.325 ± 0.11 bursts/s) compared to the period before movement onset (0.467 ± 0.12) (z=-1.540, p=0.123; 8 pairs: neg. ranks=7, pos. ranks=1). No change was seen in burst duration during ON levodopa between no-

movement (0.25 ± 0.017s) and movement (0.25 ± 0.019s) (z=-1.05, p=0.917; 6 pairs: neg. ranks=3, pos. ranks=3). Note that estimates of burst frequency and burst duration were derived from the 3 s before movement and the 1s during movement. We did not include the period from 1s to 2s after movement onset which included the post-movement synchronization. Overall, the findings are consistent with (but by no means prove) the hypothesis that for successful voluntary movement to occur burst frequency and duration must be attenuated in patients OFF levodopa, bringing beta burst characteristics more in line with those seen ON levodopa. Example shown in **A** corresponds to subject 8, right hemisphere. Values are represented as mean + SEM; *$p_{uncoorr}$<0.05, **$p_{uncoorr}$<0.05;

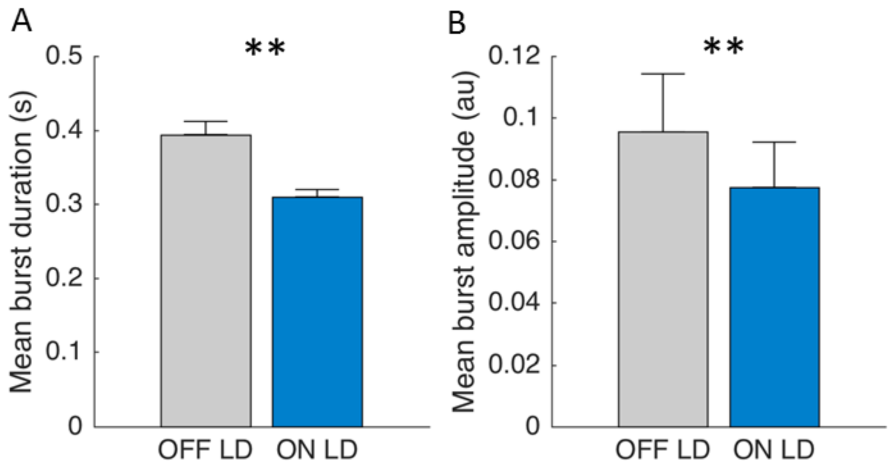

Supplementary figure 9: Relaxed definition of beta bursts. Shows the mean burst duration **(A)** and mean burst amplitude **(B)** for OFF and ON levodopa derived with a less rigorous definition of beta burst. Instead of bursts being defined according to their beta peak frequency only, here bursts are defined using a broader frequency range (beta peak frequency ± 5Hz). Importantly, although the burst definition is relaxed, there are still consistent results with reduced burst duration and burst amplitude during the ON levodopa state. Beta bursts were determined using the 75th percentile amplitude threshold. Values are represented as mean + SEM; **p<0.01.

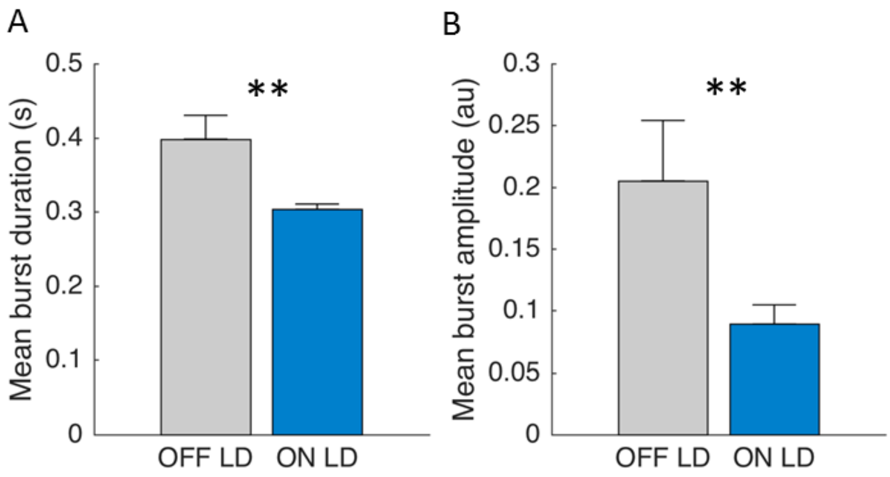

Supplementary figure 10: Beta bursts when using individual threshold. Shows the mean burst duration **(A)** and mean burst amplitude **(B)** for OFF and ON levodopa from beta bursts defined using an individual 75th amplitude threshold. Importantly, although using a separate threshold the results are still consistent with a reduced burst duration (t_{15}= 3.37, p= 0.004) and burst amplitude (t_{15}=2.84, p=0.012) during the ON levodopa state. Values are represented as mean + SEM; *p<0.05, **p<0.01.

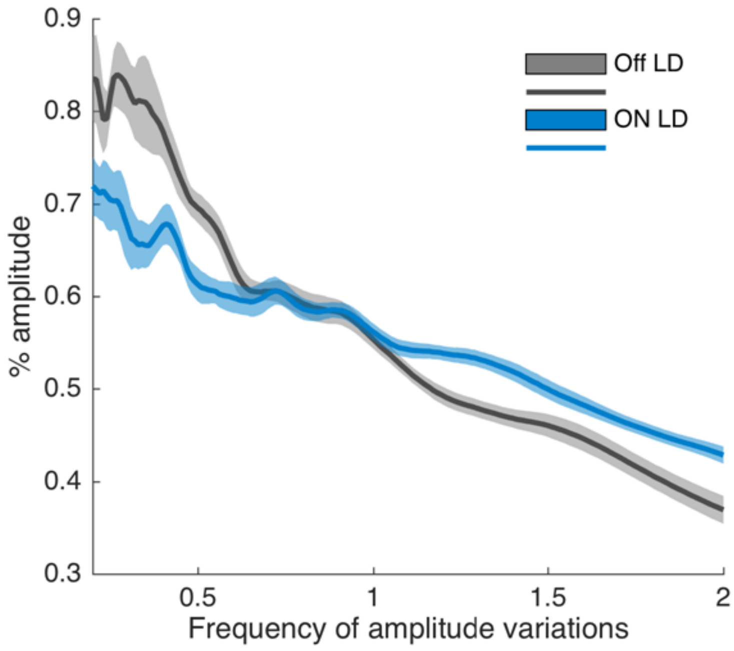

Supplementary figure 11: Amplitude variability. Illustrates the results of the frequency decomposition of the amplitude envelope of the peak beta activity for both OFF levodopa and ON levodopa. During OFF levodopa the beta amplitude envelope shows a more pronounced low frequency variation compared to ON levodopa in which amplitude variations have a relative higher frequency. These results were obtained without setting any amplitude threshold and are consistent with longer beta bursts during OFF levodopa and shorter beta bursts during ON levodopa. Values are represented as mean ± SEM (shaded areas).

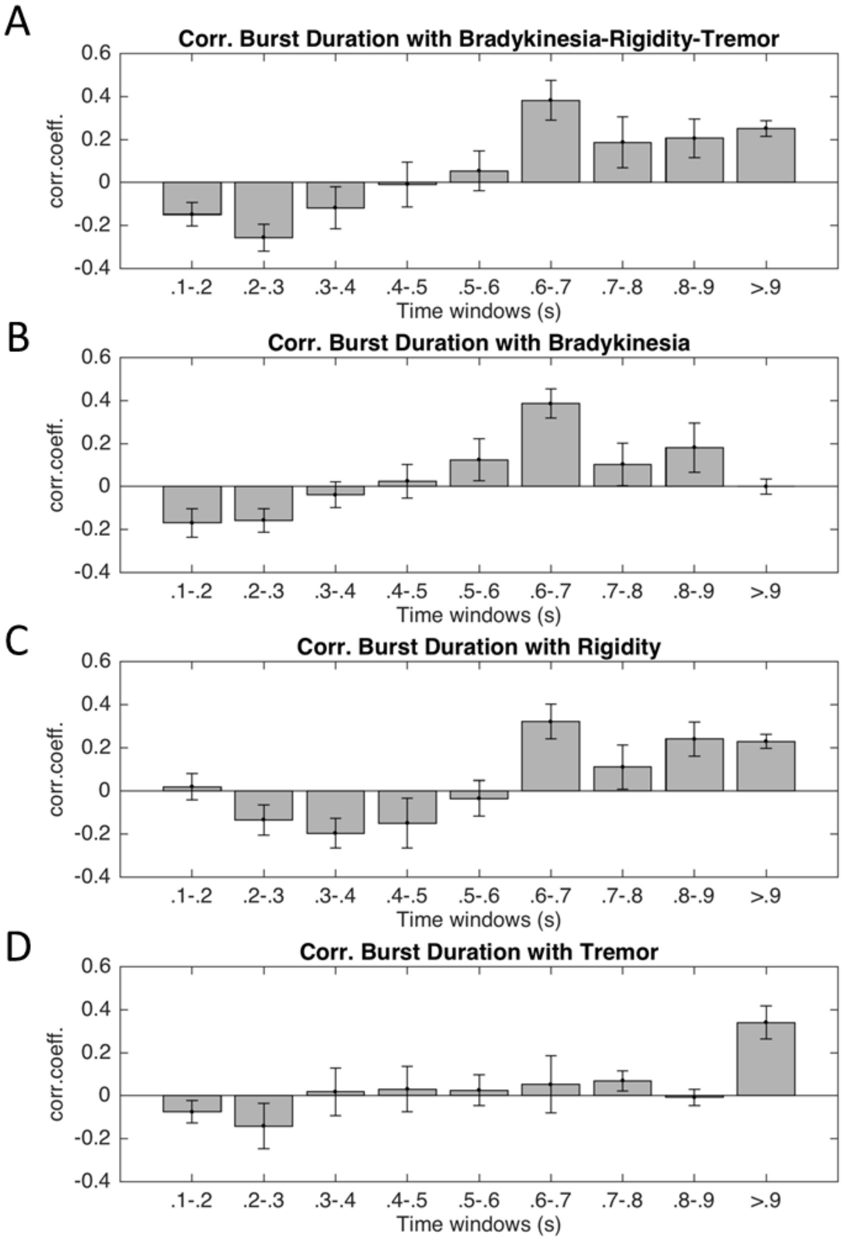

Supplementary figure 12: Correlation between motor scores and burst duration. Illustrates the mean (±SEM) Fisher transformed Spearman's correlations between clinical impairment (**A:** total UPDRS items 20-26, see also figure 6; **B:** bradykinesia, items 23-26; **C:** rigidity, item 22; **D:** tremor, items 20-21) and the percentage amount of bursts of different durations during the OFF levodopa condition. Calculations were repeated for various percentile amplitude thresholds (55-90 percentile) and then averaged. A higher amount of shorter bursts tend to be negatively correlated with clinical impairment and a higher amount of longer bursts tend to be positively correlated with clinical impairment. For the tremor items the trend is clearly weaker compared to bradykinesia and rigidity items, although still present.

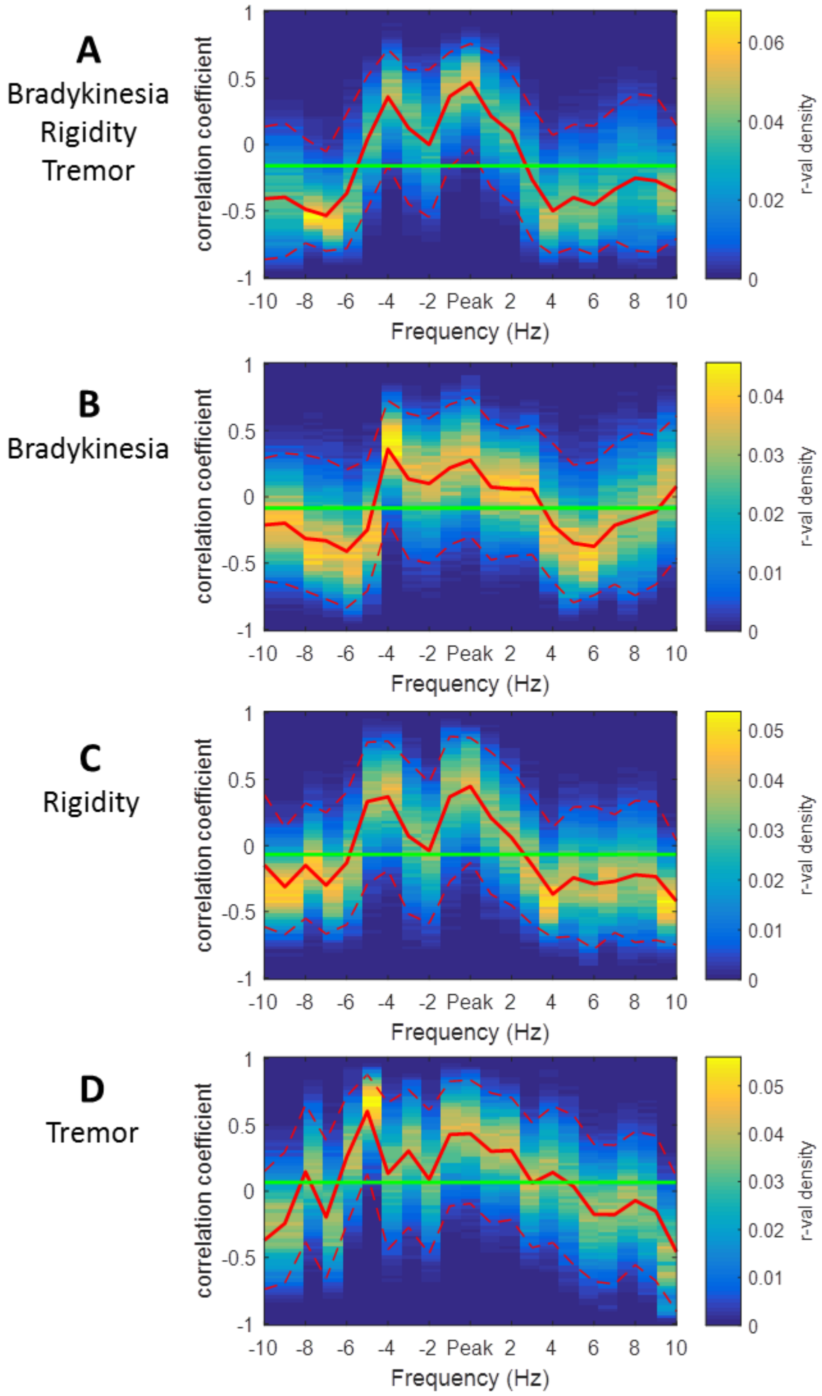

Supplementary figure 13: Correlation between motor scores and change in burst duration. Shows the Spearman's r-values of the correlation between the ratio of median burst durations between the conditions and the motor improvement in contralateral motor scores across hemispheres and patients (**A:** total UPDRS items 20-26, see also figure 6; **B:** bradykinesia, items 23-26; **C:** rigidity, item 22; **D:** tremor, items 20-21) at the beta peak frequency ± 10Hz frequency bins. For all conditions there is a positive relationship between the decrease in burst duration and clinical improvement, which is centred around the individual beta peak frequency. For the tremor sub items the relationship is weaker although

still present. The horizontal green line illustrates the mean r-value, the red dashed lines show the 95 confidence limits of the r-value density distribution of 10,000 bootstrap cycles (bootstrap method, see Methods section).
